# Cross-Scale Interactions and the Distribution-Abundance Relationship

**DOI:** 10.1371/journal.pone.0097387

**Published:** 2014-05-29

**Authors:** Earl E. Werner, Christopher J. Davis, David K. Skelly, Rick A. Relyea, Michael F. Benard, Shannon J. McCauley

**Affiliations:** 1 Department of Ecology and Evolutionary Biology, University of Michigan, Ann Arbor, Michigan, United States of America; 2 School of Forestry & Environmental Studies, Yale University, New Haven, Connecticut, United States of America; 3 Department of Biological Sciences, University of Pittsburgh, Pittsburgh, Pennsylvania, United States of America; 4 Department of Biology, Case Western Reserve University, Cleveland, Ohio, United States of America; 5 Department of Biology, University of Toronto Mississauga, Mississauga, Ontario, Canada; University of Calgary, Canada

## Abstract

Positive interspecific relationships between local abundance and extent of regional distribution are among the most ubiquitous patterns in ecology. Although multiple hypotheses have been proposed, the mechanisms underlying distribution-abundance (d-a) relationships remain poorly understood. We examined the intra- and interspecific distribution-abundance relationships for a metacommunity of 13 amphibian species sampled for 15 consecutive years. Mean density of larvae in occupied ponds was positively related to number of ponds occupied by species; employing the fraction of ponds uniquely available to each species this same relationship sharply decelerates. The latter relationship suggested that more abundant species inhabited most available habitats annually, whereas rarer species were dispersal limited. We inferred the mechanisms responsible for this pattern based on the dynamics of one species, *Pseudacris triseriata*, which transitioned between a rare, narrowly distributed species to a common, widely distributed species and then back again. Both transitions were presaged by marked changes in mean local densities driven by climatic effects on habitat quality. We identified threshold densities separating these population regime shifts that differed with landscape configuration. Our data suggest that these transitions were caused by strong cross-scale interactions between local resource/niche processes and larger scale metapopulation processes. The patterns we observed have relevance for understanding the mechanisms of interspecific d-a relationships and critical thresholds associated with habitat fragmentation.

## Introduction

A comprehensive theory of ecological communities enabling prediction of both species distributions and their relative abundances remains elusive. For ecologists interested in the assembly of communities, studies of distribution inevitably have taken inspiration from the niche theoretic framework (e.g., [1], [2]). As Hubbell [3] has noted, however, this tradition is nearly silent on the question of the relative abundances of species. In contrast, there is a rich tradition exploring patterns in relative abundances of species in a community [4], [5], [6], but this body of work has contributed little to a framework for predicting species' distributions. Nonetheless, strong empirical relationships between some measure of local abundance and regional distribution of species have been reported in a number of different contexts (e.g., core-satellite hypotheses, [7], distribution-abundance relationships, [8], and predictions from metapopulation biology, [9]), suggesting that strong linkages intertwine both distribution and abundance. In fact, distribution-abundance (also abundance-occupancy) relationships are one of the most widely observed macroecological patterns, and have been touted as one of the most general patterns in ecology [8] with roots at least back to Darwin [10]. A positive interspecific relationship between local abundance and regional distribution has been documented in a great variety of taxa including protists, plants, insects, amphibians, fish, birds, and mammals [8], [11], [12], [13]. Reviews (e.g., [8], [13]) indicate that this relationship is robust interspecifically for a wide range of different taxa across multiple scales even when abundance and distribution are estimated in different ways.

As many as thirteen (not mutually exclusive) hypotheses have been offered on the mechanisms underlying the distribution-abundance relationship (hereafter d-a relationships, [8], [13], [14]). Hypotheses range from those attributing the relationship to artifacts of sampling, phylogenetic non-independence, resource use, the spatial coupling of populations, or self-similarity and neutral models. For example, Brown [15] posited that species with broader niches are able to achieve higher local densities and inhabit a broader array of habitats (see [16] for the translation to a vital rates argument). Thus, variation in habitat quality over time or space can affect both average local abundances and the range of suitable habitats leading to a relation between density and distribution. The spatial coupling hypotheses are framed primarily around metapopulation processes, i.e. increases in local densities both by increasing dispersal success (rates of colonization and rescue/mass effects) and reducing rates of extinction enhance patch occupancy rates leading to a relation between distribution and abundance [9], [17]. Models incorporating species-level self-similarity also can generate d-a relationships (e.g., [18]). Few of these hypotheses, however, have received substantive support and there is little consensus regarding the mechanistic basis of d-a relationships [13], [19].

Though often acknowledged that it is unlikely that any of these hypotheses will be supported to the exclusion of the others [12], [13], there nevertheless has been little progress in understanding how interactions of these processes might influence the d-a relationship. The mechanisms presumed to underlie the d-a relationship are descriptions of processes operating at different scales, and affecting distribution and abundance in different manners (e.g., [13], [20]). Thus, it is likely that interactions of processes across these scales largely determine the d-a relationship and that exploration of these interactions will provide insight on why species are positioned differently on the relationship. Further, the proposed mechanisms largely assume a single-species perspective (i.e., dynamics of a species is assumed to be independent of others, e.g., [13]) although this assumption is not always made explicit. In many if not most cases, however, the species included in a d-a relationship are elements in larger metacommunities where one would assume that both local species interactions and regional processes contribute to community organization and therefore species densities and the range of habitats occupied.

We hypothesized that d-a relationships largely emerge from strong cross-scale interactions between processes associated with niche hypotheses (dynamics of species interactions influencing local densities) and those embodied in the metapopulation hypotheses (colonization and extinction/dispersal mechanisms), and that there would be important feedbacks between local density and regional distribution. Theory predicts that such cross-scale interactions involving nonlinear processes can often lead to large and unpredictable changes in distribution and abundance (e.g., [21]), or rapid regime shifts in system characteristics [22], [23]. Thus, we predicted that species would exhibit marked shifts between the states of rare and narrowly distributed versus common and broadly distributed.

We tested these hypotheses by examining the intra- and interspecific d-a relationships for a metacommunity of 13 species of pond-breeding amphibians inhabiting 37 ponds. We first determined if an interspecific d-a relationship existed for this metacommunity. We then tested the hypothesis that accounting for the number of suitable habitats for each species would cause the d-a relationship to increase rapidly then sharply decelerate. Such a relationship would suggest that species were divided into two groups, those on the slope that were dispersal limited and those on the flatter portion of the relation that were not. We posited that as a consequence these two groups of species would differ in 1) connectivity, 2) correlations between available and occupied habitats, and 3) intraspecific d-a relationships, and tested these hypotheses by comparing species groups. Our inquiry into mechanisms responsible for these groups was aided by the impacts of a drought on local habitat quality (i.e., a spatially correlated extrinsic factor or Moran effect, [24]), which caused one species to undergo wide changes in distribution and abundance over the sampling period. This Moran effect enabled us to more specifically test hypotheses that both local habitat quality/population growth rate (niche) and dispersal (metapopulation) processes affected changes in distribution and abundance, and that their interaction and feedbacks across scales generated regime shifts associated with local density thresholds. Finally, we argue that dynamics of this species were consistent with patterns in the whole community interspecific d-a relationship and provide insight into the mechanisms generating d-a relationships.

## Methods

### Sampling protocol

We estimated larval densities of 14 species of amphibians in 37 ponds on the University of Michigan's E. S. George Reserve (hereafter ESGR) over 15 yrs (1996 to 2010). The ESGR is a 525 ha tract located 40 km northwest of Ann Arbor, Michigan (42°28′N, 84°00′W). Sampling was conducted the third week in May and the third week in July to estimate densities of spring and summer breeding species. Details of the sampling protocol can be found in Werner et al. [25], [26]. In brief, samples were taken by “pipe sampling” [27], dipnetting and seining. The pipe sampler was constructed of a 76-cm length of 36-cm diameter aluminum pipe that sampled 0.1 m^2^ of water column and sediments. The sample was taken by quietly approaching an area and quickly thrusting the pipe through the water column and into the sediments to seal the sample area. Nets were then employed to remove all animals from the sampled water column and the first few centimeters of the sediments (see [28] for an evaluation of the technique).

Sampling effort varied with size of the pond typically ranging from 20–40 pipe samples haphazardly distributed across microhabitats in the ponds. Following completion of the pipe sampling, we dipnetted the pond for the person-minutes equivalent to the number of pipe samples taken. In deeper ponds (5 water bodies) the standard sampling was supplemented with two hauls of an 8-m bag seine in the deeper areas. Pond characteristics were also estimated, including area, forest canopy cover over the pond, and pond hydroperiod (fraction of days the pond held water between late March and the end of October, see [25], [26]).

We included 13 of the 14 amphibian species in analyses; the American toad (*Bufo americanus*) was excluded as it schools as larvae (e.g., [29]) and thus is highly aggregated in small sections of ponds. Consequently we do not sample this species as well as the others and our density estimates are not representative of overall pond density. Most species were sampled roughly midway through the larval period at a mean Gosner stage of 28–36 (spring breeders in May and summer breeders in July). The bullfrog (*Rana catesbeiana*), and green frog (*R. clamitans*) were exceptions; these species breed later in the summer and typically overwinter as larvae, metamorphosing in June/July of the second summer. As a consequence these species were sampled just after hatching (mean Gosner of 25) in July or as second-year individuals in May (mean Gosner of 35–36), in either case biasing density estimates relative to the other species. We therefore adjusted density estimates for these two species by estimating survivorship of cohorts across all ponds from the first to second year age classes (0.20±0.04%) and adjusted densities to the mean survivorship at the midpoint between year 1 and 2 age classes. We assigned presences to the year of larval hatching to make the presence data comparable with other species (all of which emerge the same summer as eggs are laid).

### Interspecific d-a relationship

We first examined the d-a relationship across all species; data were average densities in occupied ponds and average number of ponds occupied by a species over the 15 yrs. We computed average local density for each species by taking the mean density in occupied ponds each year and then averaging these values across years (hereafter simply mean density). If a species was sampled in both May and July, we employed the date with the maximum density estimate. It is expected that the d-a relationship will saturate (depending on grain size, [13]; therefore we fit the data to the following nonlinear relationship, y = a(1−b^x^)).

We tested whether the d-a relationship was due to phylogenetic inertia by regressing phylogenetic independent contrasts [30] for occupancy onto phylogenetic contrasts for abundance. We compiled a composite phylogeny of the species based on known, uncontroversial phylogenetic relationships [31], [32], [33]. The relationships among species were well resolved in two large phylogenies except for the relationships among the three species of *Ambystoma* and the relationship of the wood frog (*Rana sylvatica*) to the other ranid frogs. Thus, in our composite phylogeny we used the well-supported relationships among *Ambystoma* from Shaffer et al. [33] and the well-supported relationship of wood frogs to other ranid frogs from Hillis and Wilcox [32]. We used the package “ape” in the program R (version 2.8.0, [34]) to calculate phylogenetic independent contrasts for distribution and abundance.

### The constrained interspecific d-a relationship

In addition to examining the relationship between average local density and average number of ponds occupied, we computed a “constrained” d-a curve, i.e. the relationship between average local density and the average fraction of potentially habitable ponds occupied (i.e. of ponds estimated to be uniquely available to each species). We estimated the range of potentially habitable ponds from the cumulative list of ponds in which a species was recorded over the 15-yr monitoring period. For most species the cumulative curve of ponds occupied approaches an asymptote after 3 to 5 yrs of sampling (Figure S1, Appendix S1).

For each year we then further refined this list of potentially habitable ponds by including only those ponds available for breeding to that species (and that did not dry before sampling). We considered ponds available for breeding each year to be those with a mean pond condition >30% for the 30 days surrounding the peak of each species' breeding season (based on our call surveys, see [25], and literature accounts). Pond condition was defined as maximum area of the pond that year during this 30-d breeding period divided by the maximum area ever recorded in the pond (bathymetric data on ponds enabled calculation of pond areas from depth gauge measurements). Analyses over the 15 yrs. indicated that breeding for all species dropped off precipitously if this pond condition factor was <30% (Werner et al. unpublished data).

If species experienced no dispersal limitation, the null expectation for the constrained d-a curve would be a line with slope of 0 and occupancy values near 1 (i.e., all sites appropriate for a species would be occupied regardless of local density). If species were strongly dispersal limited on the ESGR over the entire monitoring period we would underestimate the (cumulative) number of ponds suitable for a species, but this underestimate should bias conclusions in the direction of the null expectation. To determine the shape of this constrained relationship, we fit the data to linear, nonlinear (y = a[1−b^x^]), and segmented linear (piecewise) regression models (see *Threshold analyses*), and employed AIC_c_, a bias-corrected version of Akaike's information criterion, to rank models according to the strength of support from the data [35].

### Intraspecific d-a relationships

In order to examine temporal trends for each species over the 15 yrs, we also examined the intraspecific d-a relationships, i.e. mean density of the species in occupied ponds each year plotted against the number of ponds occupied that year.

### Connectivity analyses

We employed several indices to assess the connectivity of ponds for different species; connectivity was assessed using either a resistance metric based on electric circuit theory [36] or the Hanski connectivity index (reviewed in [37], [38]). The former metric provides a distance weighted according to the estimated permeability of the landscape separating populations including multiple potential routes between ponds, whereas the latter accounts for spatial position and population densities in ponds but is independent of intervening terrestrial habitat characteristics. Details of these methods are presented in Appendix S2.

### Threshold analyses

In order to test for thresholds in d-a relationships, we employed segmented linear (piecewise) regression with a break-point (e.g., [39]). Analyses were conducted with segmented regression software [40], which introduces breakpoints and calculates separate linear regressions for each segment. The optimal breakpoint is that with the smallest confidence interval. The value of introducing breakpoints is evaluated by assessing whether the piecewise regression models perform significantly better than linear regression models. The program selects the function type that maximizes the coefficient of determination (R^2^), and passes a test of significance based on an alpha value of 0.05 (degrees of freedom are corrected down appropriately as number of parameters increases with the different models [41], [42]). In addition, we assessed whether model selection by these criteria was consistent with that employing the bias-corrected version of Akaike's information criterion.

### Cross-correlations

To evaluate whether species differed in how closely they tracked changes in available habitats, we computed correlations between time series of the number of ponds available to each species (as estimated above) and the number of ponds actually occupied each year.

### Chorus frog dynamics: regime shifts

The chorus frog (*Pseudacris triseriata*) exhibited substantial dynamics over the monitoring period allowing us to consider the transition between rarity and commonness. To determine if there were discrete shifts in its pond occupancy or density, we tested for change points or regime shifts in the time series [43]. We employed the exploratory or data-driven analysis of Rodionov [44], [45] based on sequential application of Student's t-test (null hypothesis that the nth observation is drawn from the same population as the preceding sequence). Rejection of the null identifies a potential regime shift that is then finally confirmed or rejected based on subsequent observations (algorithm available at http://www.beringclimate.noaa.gov/regimes/). Unlike most methods, performance does not deteriorate if change points are near the end of the series. We employed two-tailed tests with an α-level of 0.05 unless noted otherwise and we varied the expected regime lengths between 3 and 10 years. Regimes longer than the specified cut-off length are all detected, whereas those shorter will likely only be detected if highly significant [44], [45].

### Availability of suitable habitats for the chorus frog

We sought more refined criteria than the cumulative ponds occupied to categorize ponds as suitable to support a larval population of chorus frogs when it was rare on the landscape. We know from experimental and survey data that chorus frog performance is substantially better in open- than in closed- (>75% cover) canopy ponds [25]. Its performance is also better in temporary than in permanent ponds (see [27], [46], [47], [48]). We predicted that occupancy and density would decline significantly from 1) open-canopy ponds that dried the previous fall, to 2) open-canopy ponds that did not dry the previous fall, and 3) closed-canopy ponds. We evaluated this categorization with our data from the period when regional populations of the chorus frog were extremely large and the species did not appear to be dispersal limited. We calculated the frequency of chorus frog presences for the three pond categories over this period and the average density achieved when present.

As a first approximation of the number of suitable habitats when the chorus frog was rare, we tabulated the number of ponds in each of the above three categories each year, weighted them by the frequency of chorus frog occupancy when abundant, and summed these values. Rounding to the nearest integer provided an expected number of ponds available to the chorus frog assuming little or no dispersal limitation, and this value was compared to actual occupancy values.

### Ethics statement

This study was carried out in strict compliance with the recommendations in the Guide for the Care and Use of Laboratory Animals of the National Institutes of Health. The protocol was approved by the University Committee on Use and Care of Animals of the University of Michigan (Permit Number: 07765). Collectors permits for field work were approved by the Aquatic Species Regulatory Affairs Unit, Fisheries Division, of the Michigan Department of Natural Resources.

### Data availability

The data employed in the manuscript are available on Dryad (doi:10.5061/dryad.js47k).

## Results

### Interspecific d-a relationship

**Figure 1:**
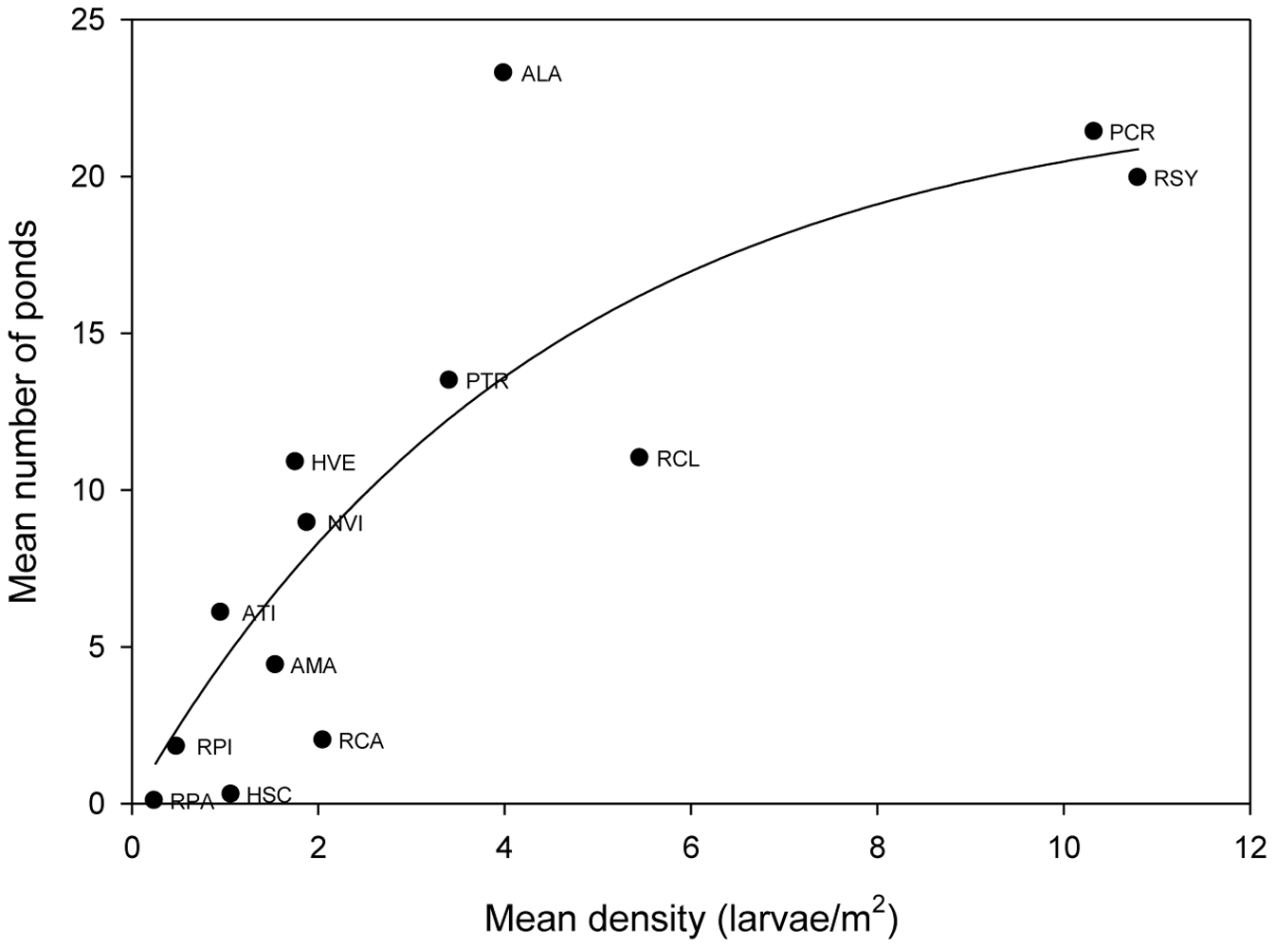
Interspecific distribution-abundance relationship for thirteen species of ESGR amphibians. Data fit to the relationship, y = a(1−b^x^), R^2^ = 0.73, F_1,11_ = 30.4, p = 0.0002. Species represented are: *Hyla versicolor* (Hve), *Pseudacris crucifer* (Pcr), *P. triseriata* (Ptr), *Rana catesbeiana* (Rca), *R. clamitans* (Rcl), *R. pipiens* (Rpi), *R. sylvatica* (Rsy), *Ambystoma laterale* (Ala), *A. maculatum* (Ama), *A. tigrinum* (Ati), and *Notophthalmus viridescens* (Nvi).

There was a highly significant d-a relationship among the ESGR amphibians (Figure 1). Further, there was a significant positive relationship between the phylogenetic independent contrasts of distribution and abundance (slope = 1.92±0.40, t = 4.76, p<0.0001), indicating that the relationship was not an artifact of phylogenetic history.

### The constrained interspecific d-a relationship

**Figure 2:**
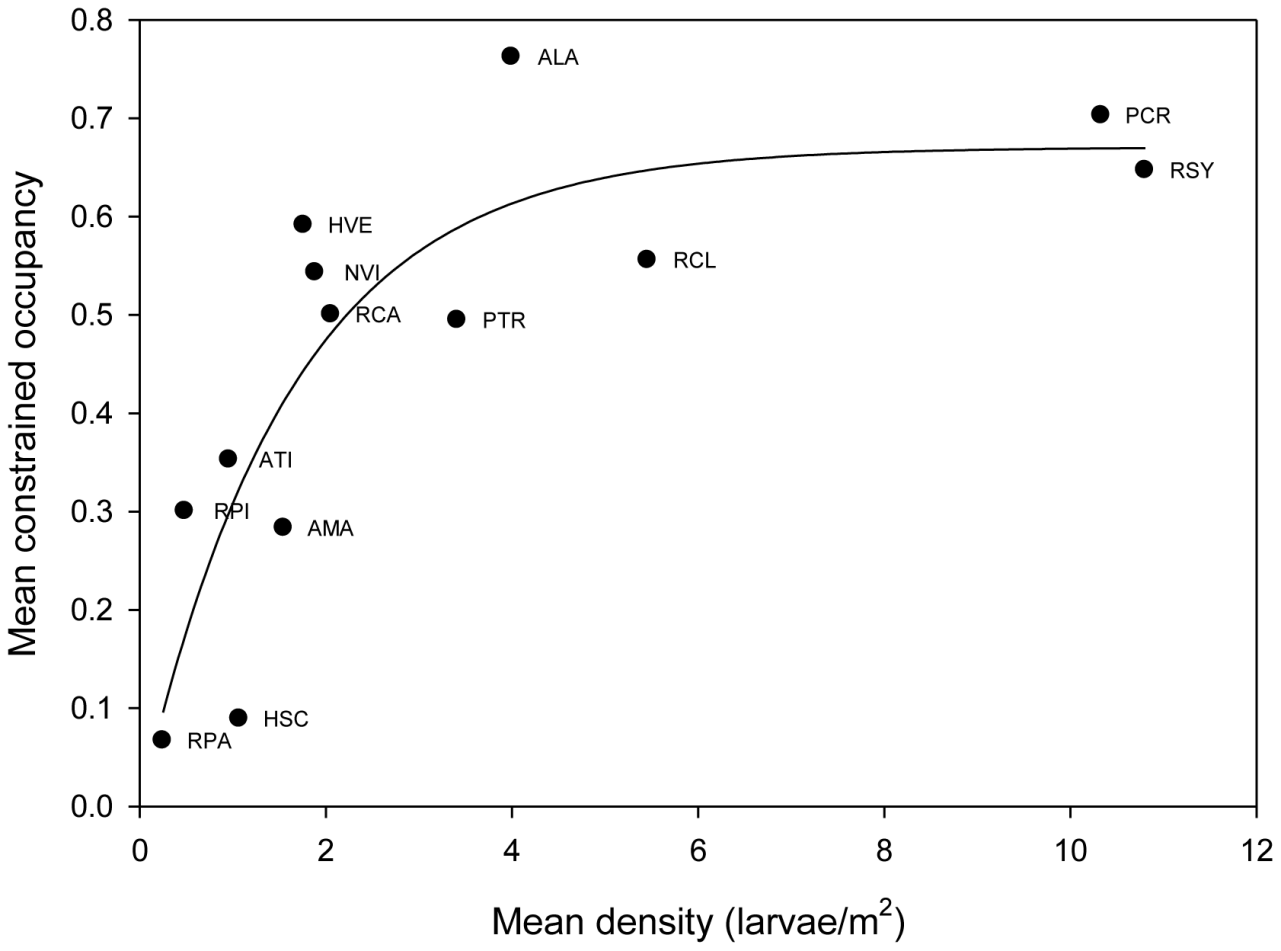
Constrained interspecific distribution-abundance relationship for the ESGR amphibians. The dependent variable is the average fraction of potentially habitable ponds occupied fit to the relationship, y = a(1−b^x^), R^2^ = 0.70, F_1,11_ = 28.6, p = 0.0002. Species designations as in Figure 1.

The constrained d-a relationship, which included only those ponds deemed habitable, rose sharply and then flattened out (Figure 2). The best supported model was the nonlinear model, but there was also strong support for a segmented regression of two horizontal lines, one with a mean occupancy of 0.60 and one with a mean occupancy of 0.22, with the break occurring at a density of 1.6 individuals/m^2^ (Table 1, the latter was also identified as the best model by the segmented regression algorithm). In either case the inference is similar, i.e., 8 of the 13 species lie on or near the upper portion of the relationship occupying a relatively constant fraction of the ponds that they had historically inhabited. Of the 5 species found lower on the curve, two of them were too rare in our samples for further analysis (pickerel frogs *[R. palustris]*) and four-toed salamanders *[Hemidactylum scutatum]*). The three remaining species, spotted salamander (*Ambystoma maculatum*), tiger salamander (*A. tigrinum*), and leopard frog (*R. pipiens*), had constrained occupancy values that were considerably lower than the 8 species above (ranging between 28 and 35%). For convenience, hereafter we will refer to these two groups of species as rare and abundant species.

**Table 1:** Model selection analysis for the constrained distribution-abundance relationship*.

Model	K	N	Residual SS	AIC	AIC_c_	Δ_i_	Exp (−Δ_i_/2)	W_i_	Evidence Ratio
Nonlinear [f = a(1−b^x^)]	3	13	0.16	−51.06	−48.40	0.00	1.000	0.692	1.00
Horizontal/horizontal	4	13	0.13	−51.67	−46.67	1.73	0.423	0.292	2.37
Linear [f = a+bx]	3	13	0.32	−42.36	−39.70	8.70	0.013	0.009	77.52
Sloping/horizontal	5	13	0.15	−47.67	−39.09	9.30	0.010	0.007	104.73
Mean	2	13	0.58	−36.47	−35.27	13.13	0.001	0.001	708.16
Sloping/sloping	6	13	0.16	−45.25	−31.25	17.15	0.0002	0.000	5288.63

* Linear, nonlinear, and various segmented regression models were compared employing AIC_c_. For example, sloping/horizontal refers to a linear fit of a sloping line to the data up to the breakpoint and then a horizontal line after the breakpoint.

We posit that the eight abundant species (occupying a relatively constant fraction of estimated available ponds) conform to a simple habitat-filling model (i.e., experience little dispersal limitation and colonize most suitable habitats as they become available, [16], [49]). In contrast, we hypothesize that the rare species were more likely dispersal limited. The following three predictions would be consistent with this interpretation: 1) ponds containing rare species should be significantly more connected compared to those where these species were absent, whereas this would not be the case with abundant species, 2) pond occupancy of abundant species should closely track changes in availability of ponds, whereas this would not be the case with rare species, and 3) no intraspecific relation between density and pond occupancy should exist for abundant species, whereas rare species should exhibit a positive d-a relationship if occupancy or density varied substantially over the study period.

Prediction 1. We compared rare species to abundant congeners contrasting the connectivity of all ponds for each species pair. This analysis assumed years were independent data points (average of the autocorrelations for the seven species was 0.24±0.04, on average the connectivity of a pond the previous year only explained 7±2% of the variation in next year's connectivity). Employing the Hanski index, ponds occupied by tiger and spotted salamanders (rare species) were significantly more connected than unoccupied ponds (spotted: t (62) = 2.22, p = 0.03, tiger: t (99) = 2.0, p = 0.047). This was not the case for the blue-spotted salamander (*A. laterale*), an abundant congener (t (479) = −0.12, p = 0.91). Spotted and tiger salamanders exhibited population structures suggestive of island/mainland metapopulations with several source ponds apparently supplying colonists to a number of highly connected ponds that exhibited sporadic presences (see Appendix S2).

We observed a similar pattern for the two spring-breeding ranid frogs; employing the Hanski index, ponds occupied by the rare leopard frog were significantly more connected than unoccupied ponds (t (25) = 3.5, p = 0.002), whereas the abundant wood frog showed no difference (t (479) = −0.03, p = 0.98). This trend also was consistent contrasting the abundant spring peeper and chorus frog during years when rare (1997–2001, see further discussion below), although connectivity differences were not significant for either species. Before expansion, ponds occupied by chorus frogs exhibited an average connectivity 1.4 times that of unoccupied ponds, whereas spring peeper connectivity trended in the opposite direction, i.e. higher (1.1 fold) for unoccupied ponds. After chorus frog expansion, its pattern altered such that unoccupied ponds were 1.3 times more connected than occupied ponds (i.e., most suitable ponds are occupied and those not suitable are embedded in the matrix).

**Figure 3:**
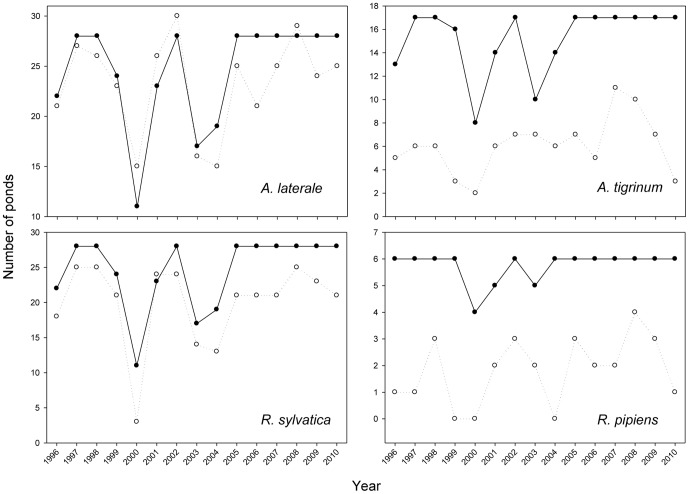
Temporal trends of ponds occupied (open symbols, dashed line) versus available (closed symbols, solid line). Comparisons for congeners from two families (*Ambystoma* and *Rana*) where one species was abundant (left column, i.e. on the flat portion of the constrained distribution-abundance curve) and one rare (right column, i.e. low on the curve).

Prediction 2. Cross-correlations of estimated ponds available to each species for breeding and the number of ponds occupied indicated that abundant species exhibited significantly higher correlations than the rare species (mean r's of 0.74 and 0.40 respectively, t (7) = 2.58, p = 0.036). Four of the six abundant species had correlations between 0.84 and 0.92 (e.g., Figure 3). This analysis excluded bullfrogs (lack of variation in ponds available precluded calculation of a correlation) and chorus frogs because they transitioned between rare and abundant (and exhibited the lowest correlation of any species, r = 0.21, p = 0.45). Further, the absolute number of occupied ponds deviated substantially less from the estimated number available for abundant species, averaging 0.61 versus 0.31 for the rare species (Figure 3, t (8) = 5.26, p = 0.001).

Prediction 3. As predicted none of the intraspecific d-a regressions were significant for the abundant species, despite wide variation in both pond occupancy rates and densities across years (Figure S3, Appendix S3). Rare species generally did not present a sufficient range in occupancy over years to adequately assess the relationship, although leopard frogs did exhibit a trend toward a positive d-a curve (linear regression, p = 0.075).

### Chorus frog dynamics

**Figure 4:**
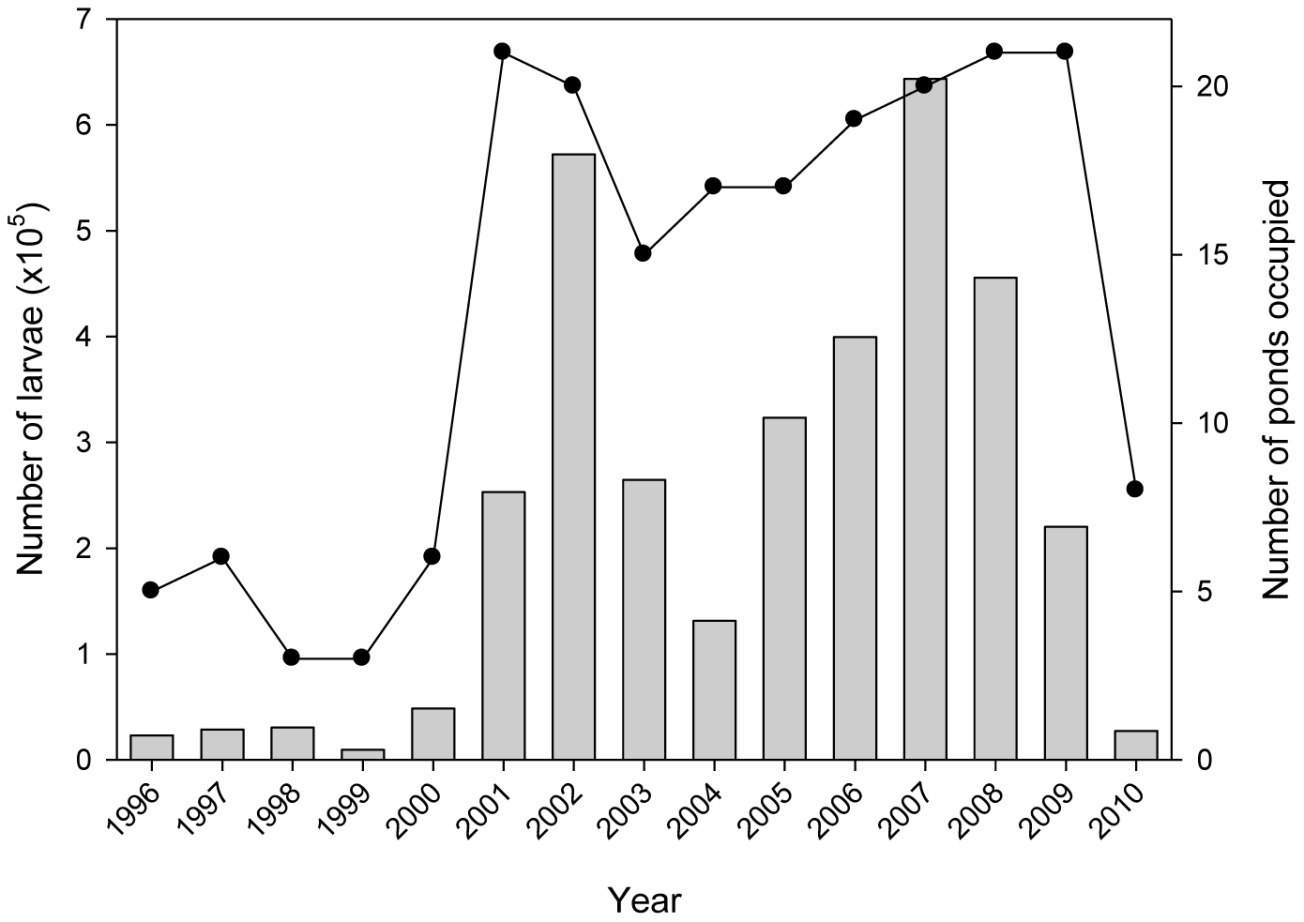
Regional population size (histograms) and number of occupied ponds (line) across years for *Pseudacris triseriata*. Regional population size was the sum across all ponds of larval population sizes (i.e., average density in a pond x pond surface area corrected for drying on each sample date).

The regional population size of the chorus frog markedly expanded and then collapsed over the study period (Figure 4). Regional population size grew nearly exponentially between 1999 and 2002; in 2000 there were 6 extant populations and the following spring (2001) the chorus frog colonized 15 new ponds [48]. Regional population sizes remained high with the exception of 2004 (when late spring drying prevented or reduced oviposition in a number of ponds) until 2007 when population size declined precipitously through 2010. Pond occupancy was high between 2001 and 2009, but between 2009 and 2010 the number of occupied ponds collapsed from 21 to 8. Thus, the chorus frog transitioned between a rare species (the first five years it occupied 22.3% of estimated available ponds) to an abundant species (the next five years it occupied 84.4% of estimated available ponds), suggesting a potential regime shift in population structure.

Significant change points in pond occupancy were detected at the 2000–2001 boundary and at the 2009–2010 boundary (Figure 4, Table 2). These shifts were significant for all expected regime lengths between 3 and 10 years, indicating a very robust deviation in mean values of regimes [45]. Shifts in ln mean density were not as dramatic; we conducted the initial search at an α-level of 0.1 and detected significant shifts for expected regime lengths of 4 and 5 years at the 1999–2000 boundary (actual p value = 0.016) and the 2009–2010 boundary (Table 3). Mean density of chorus frog larvae (#/m^2^) in occupied ponds averaged 1.7 between 1996 and 1999, but 4.3 between 2000 and 2009, and then dropped back to 1.7 in 2010. Thus, overall there was strong evidence of a marked transition in population structure of chorus frogs at the landscape level reflected in both occupancy and density. These analyses indicated that the change point in chorus frog pond occupancy lagged that in mean local density by a year (also evidenced by the cross-correlation of ln mean density and occupancy which was maximized with a lead of one year on density, r = 0.61, p = 0.02).

**Table 2:** Rodionov regime shift analysis of pond occupancy for the chorus frog*.

Year	Occupancy	RSI	Mean	Length	Confidence
1996	5	0	4.6	5	
1997	6	0	4.6	5	
1998	3	0	4.6	5	
1999	3	0	4.6	5	
2000	6	0	4.6	5	
2001	21	1.0147	19	9	1.44E-08
2002	20	0	19	9	
2003	15	0	19	9	
2004	17	0	19	9	
2005	17	0	19	9	
2006	19	0	19	9	
2007	20	0	19	9	
2008	21	0	19	9	
2009	21	0	19	9	
2010	8	−0.5700	8	1	

*Regime shift analysis for pond occupancy (0.05 level, expected regime length of 4 years). RSI is the regime shift index [44], and mean and length are mean number of ponds occupied in a regime and the length of the regime. If a regime shift is detected at a pre-determined α-level and expected regime cut-off length, mean values of the old and new regimes differ statistically at least at the given level (actual significance level is calculated for shifts with a number of preceding and following years, e.g., 2000–2001 occupancy boundary differed at the 1.4E-08 level, [44], [45]).

**Table 3:** Rodionov regime shift analysis of density for the chorus frog*.

Year	Density	RSI	Mean	Length	Confidence
1996	0.262	0	0.485	4	
1997	0.034	0	0.485	4	
1998	0.693	0	0.485	4	
1999	0.949	0	0.485	4	
2000	1.966	0.4367	1.324	10	0.016
2001	1.030	0	1.324	10	
2002	2.055	0	1.324	10	
2003	1.351	0	1.324	10	
2004	0.095	0	1.324	10	
2005	1.133	0	1.324	10	
2006	1.165	0	1.324	10	
2007	1.672	0	1.324	10	
2008	1.655	0	1.324	10	
2009	1.115	0	1.324	10	
2010	0.523	−0.0678	0.523	1	

*Regime shift analysis for ln mean density (0.1 level, expected regime length of 4 years). See Table 2 for details.

### Factors contributing to chorus frog dynamics at different scales

An ENSO-related drought beginning in the fall of 1998 and extending through the fall of 2006 greatly enhanced pond drying on the ESGR [48]. For example, the proportion of ponds that dried the prior fall was 0.36±0.07 before and after the drought (1996–1998, 2008–2010) but this proportion more than doubled to 0.80±0.03 during the drought (1999–2007). Mean hydroperiod across all ponds, measured as the percent of days a pond held water between March and late October, was 92±1.4% before and after the drought (1996–1997 and 2007–2009) but only 59±3.1% during the drought (1998–2006).

Pond drying in the fall can have a major effect on predator communities the following spring when chorus frogs breed (chorus frog larvae are very active and therefore extremely vulnerable to predators, [27], [47], [50]). In spring, ESGR ponds that dried the previous fall had only 25.5% of the predator biomass of ponds that did not dry. Moreover, ponds that were also dry in the early spring and then refilled had only 14% of predator biomass of ponds that did not dry (Figures S4 and S5, Appendix S3). The most dangerous predators, fish and dragonfly larvae [51], [52], were especially affected if a pond dried the preceding fall (exhibiting <5% of the biomass of ponds that did not dry). Drying did not affect closed-canopy pond predators, likely because the important predator species in these ponds typically did not overwinter as larvae (Appendix S3).

During the years of largest chorus frog regional populations (2001 to 2008), occupancy rates averaged 93% for open-canopy ponds that dried the previous fall, 40% for open-canopy ponds that did not dry the previous fall, and 32% for closed-canopy ponds (categories differed significantly, Chi square test, χ^2^ = 50.9, p<0.001). Densities averaged 6.2, 3.0, and 0.9/m^2^ respectively in these ponds further indicating differences in pond quality (ANOVA, F_2,147_ = 8.56, p<0.001). This pattern is consistent with other studies indicating that closed-canopy ponds are sink habitats for chorus frogs presumably due to resource conditions [48]. Thus, mechanisms responsible for changes in local dynamics and availability of suitable habitats on the ESGR over the monitoring period appear clear, i.e. drying of particularly open-canopy ponds and the associated decline in predator biomass enabled both expansion of local populations and availability of new ponds on the landscape for colonization.

**Figure 5:**
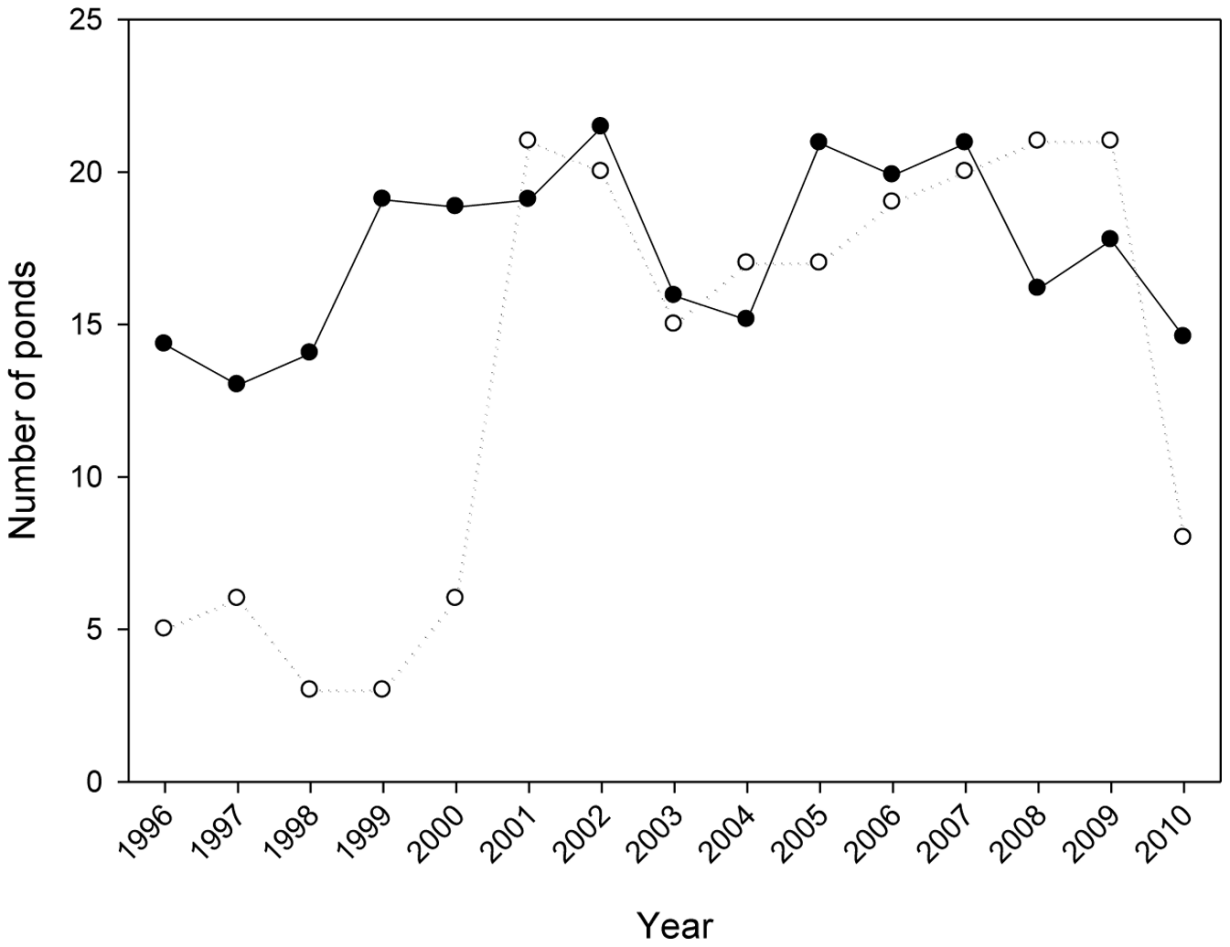
Comparison of number of ponds occupied by *Pseudacris triseriata* versus those suitable across years. Open symbols and dashed line are ponds occupied and closed symbols and solid line are number of ponds deemed suitable habitat. Ponds estimated as suitable were 1) open-canopy ponds that dried the previous fall, 2) open-canopy ponds that did not dry the previous fall, and 3) closed-canopy ponds, respectively, weighted by the frequency of occupancy in the 2001 to 2008 period, and summed (see Methods for details).

Regionally, we asked whether there was evidence of dispersal limitation when the chorus frog was rare (before and after the drought). Prior to the drought (1996 to 1998), only one-third of the ponds estimated suitable for the chorus frog (based on the above categories, see Methods) were occupied annually (Figure 5). Following initiation of the drought (fall of 1998), the number of suitable ponds rose by 40%, but only 24% of suitable ponds were occupied until 2001. Thus, there was a clear indication of dispersal limitation prior to the drought and immediately after its inception with a large number of apparently suitable ponds unoccupied.

Conversely, during the period of large regional chorus frog populations (∼2001–2008) a number of pond populations were apparently maintained by dispersal (rescue or mass effects). For example, a number of small closed-canopy ponds (sink habitats) exhibited occupancies ranging from 1 to 7 yrs. (11 ponds, 35 total presences) over the 8-yr period. Densities in these ponds averaged 0.71±0.05/m^2^ (populations averaged 685±138.2 individuals/pond). Over the 15-yr monitoring period, the probability of extinction for populations in ponds with densities <1.5/m^2^ (or populations <2500 individuals) the following year was 0.40. This rescue/mass effect carried over after the drought broke; e.g., we estimated that 17 ponds were available to chorus frogs in 2008–09 but they occupied 21 ponds, 24% more ponds than estimated available (Figure 5). However, in 2010 pond occupancy again collapsed and only 55% of ponds estimated as available were occupied. Thus, regional processes (dispersal limitation and rescue or mass effects) also appeared to be important in the dynamics of the chorus frog.

### Cross-scale interactions: threshold local densities and regime shifts

The above analyses suggest that changes in both local species interactions and regional/metapopulation processes were involved in the dynamics of the chorus frog mediated by the Moran effect of the drought. The change point analyses indicated that a shift in density preceded that in occupancy by a year, suggesting that cross-scale interactions of these processes may revolve in part around density thresholds.

The appropriate scale to investigate these interactions and threshold densities depends on dispersal distances. The ESGR ponds are largely associated with wetlands on the east and west sides of a central highland (Figure S2, Appendix S2), the extent of which considerably exceeds maximum reported dispersal distances for chorus frogs (average on the order of 100 to 150 m [48], maximum reported distances 685 m for chorus frogs [53] and 573 m for its congener the spring peeper [54]). For each ESGR pond the nearest neighbor pond fell within its respective region; average nearest neighbor distances within regions were 120.8±26.8 m (east) and 77.3±16.4 m (west), whereas average nearest neighbor distances on the opposite side of the ESGR were 1208.3±104.2 m for eastern ponds and 913±60.8 m for western ponds (limiting to ponds in which chorus frogs were recorded distances were 157.6±43.5 and 87.3±18.6 m and 1450.3±402.2 and 1257.9±324.8 m respectively). Further, for years when populations were high (2001 to 2008), there was no correlation between regional populations on the two sides (r = 0.0007, p = 0.999; populations ranged from 7,700–352,000 on the east and 87,900–220,000 on the west). Accounting for landscape context and multiple dispersal routes reinforces these analyses; using circuit theory, global mean resistance between ponds on the ESGR was always higher than that calculated for ponds on the east or west sides individually, regardless of friction values assigned to habitat types (Figure S2, Appendix S2). Therefore we might expect the two sides of the ESGR to respond somewhat independently to the Moran effect of the drought.

**Figure 6:**
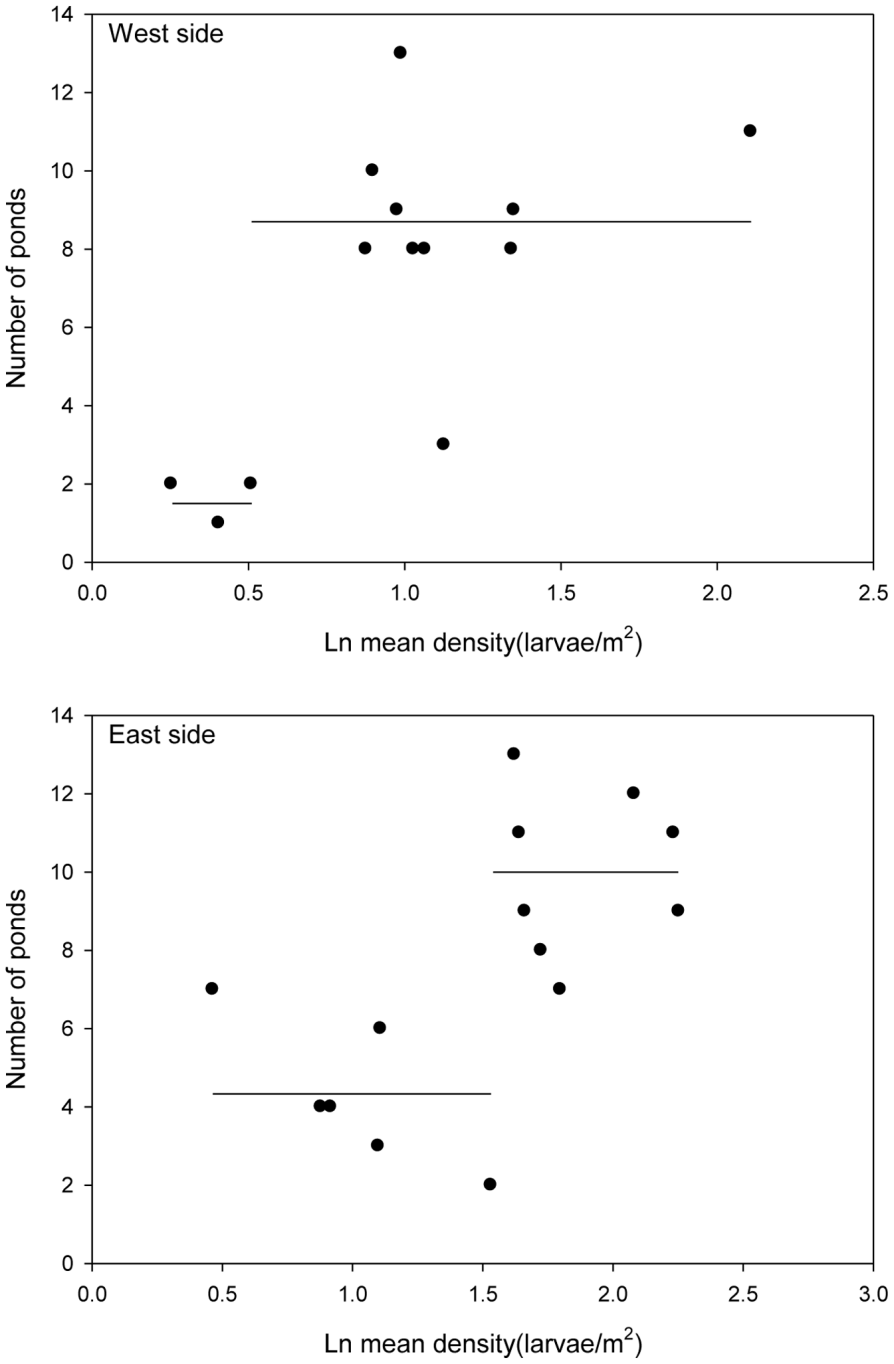
Intraspecific *Pseudacris triseriata* distribution-abundance relationships for the west and east sides of the ESGR. Relationships and thresholds between them determined by segmented regression analysis (see text for details).

Given the dynamics of the chorus frog (Figure 4), we would expect positive intraspecific d-a relationships. Linear d-a relationships were significant for both the west (ANOVA, F_1,12_ = 11.8, p = 0.005) and east sides (ANOVA, F_1,12_ = 6.8, p = 0.023, with data from the 2000 survey excluded as it was an outlier from the d-a relationship for this side primarily due to one pond (22.6 individuals/m^2^), Cook's distance = 0.83, with a cutoff value (4/n) of 0.27; this unprecedented density appears to have resulted from an abnormally low depth gauge reading that year in this pond suggesting that animals may have been concentrated by drying). However, segmented regression analysis indicated that two horizontal lines with a breakpoint provided a significantly better fit for the data on both sides (Figure 6). This was corroborated by the Akaike criterion (including the nonlinear model, Table 4). These relationships indicated significant breakpoints (thresholds) in mean densities that differed on the two sides of the ESGR, 1.67/m^2^ for the west side and 4.62/m^2^ for the east side.

**Table 4:** Model selection analyses for distribution-abundance relationships for the chorus frog*.

West side									
Model	K	N	Residual SS	AIC	AIC_c_	Δ_i_	Exp (−Δ_i_/2)	W_i_	Evidence Ratio
Horizontal/horizontal	4	14	61.1	28.63	33.07	0.00	1.00	0.62	1.00
Nonlinear [f = a(1−b^x^)]	3	14	92.6	32.45	34.85	1.78	0.41	0.26	2.43
Linear [f = a+bx]	3	14	106.0	34.34	36.74	3.67	0.16	0.099	6.26
Sloping/horizontal	5	14	68.4	32.2	39.7	6.63	0.04	0.022	27.57
Mean	2	14	209.0	41.85	42.94	9.86	0.007	0.004	138.66

East side									
Model	K	N	Residual SS	AIC	AIC_c_	Δ_i_	Exp (−Δ_i_/2)	W_i_	Evidence Ratio
Horizontal/horizontal	4	14	47.33	25.05	29.50	0.00	1.00	0.91	1.00
Linear [f = a+bx]	3	14	101.0	33.67	36.07	6.57	0.04	0.03	26.67
Nonlinear [f = a(1−b^x^)]	3	14	104.17	34.10	36.50	7.00	0.03	0.028	33.11
Sloping/sloping	6	14	36.65	25.47	37.47	7.97	0.02	0.017	53.88
Mean	2	14	157.00	37.84	38.93	9.43	0.009	0.008	111.83
Sloping/sloping	6	14	81.85	36.72	48.72	19.22	0.0001	0.000	14944.19

*Analyses for the West and East sides of the E. S. George Reserve. Model designations as in Table 1 (in the case where two sloping/sloping models are included this indicated the lines were of different orientation to each other).

Threshold densities separated regime shifts in pond occupancy; mean densities on both sides of the ESGR increased monotonically from 1998 to 2000 (but were 6.8 fold higher on the east side) and crossed unique thresholds between 1999 and 2000 precipitating colonization of numerous new ponds in 2001. Mean densities remained above threshold values from 2000–2008 (means of 9.6 and 2.5/m^2^ on east and west sides respectively), with the exception again of 2004 when the spring drying caused each to briefly dip below the thresholds. Mean densities then declined monotonically on both sides from 2008 through 2010 (2.6 fold higher on the east side) crossing unique thresholds between 2008 and 2009 and precipitating the extinctions in 2010. Thus, in both the expansion and collapse phases mean local densities crossed thresholds a year before regime shifts in occupancy.

The higher threshold density for eastern ponds was consistent with the fact that these ponds were less connected than western ponds. Western ponds largely fall along a linear array of wetlands, whereas some ponds on the east side are situated in deep depressions separating them from others (e.g., the ratios of east to west resistance values and values for their variances were always >1 if slope was accorded a resistance value of >50 and forest <30, Appendix S2). Accordingly, the eastern ponds exhibited both a higher density threshold and took longer to fully colonize (number of ponds occupied increased from 3 to 8 in 2001 and then gradually rose to a maximum of 13 ponds by 2009). The last ponds to be colonized were small closed-canopy ponds separated by steep topography from other ponds.

## Discussion

We hypothesized that strong cross-scale interactions between local (niche) and regional (metapopulation) processes are important in generating the relationship between distribution and abundance. We further hypothesized that these cross-scale interactions would prompt rapid shifts between states of rare and narrowly distributed or common and broadly distributed. The fact that species were positioned low on the constrained d-a curve or on the flat portion of the curve (Figure 2), suggested species fell into two groups, those conforming to a habitat filling model or those that were dispersal limited. Analyses of connectivity patterns, cross-correlations and intraspecific d-a relations were consistent with this interpretation. Occupied ponds for abundant species were no more connected than unoccupied ponds, and these species exhibited no relation intraspecifically between density and occupancy, despite the fact that densities varied widely across years (e.g., average spring peeper densities ranged from 3 to 26/m^2^ and wood frog densities from 3 to nearly 28/m^2^ with no effect on occupancy rates, Appendix S3). Moreover, the cross-correlations indicated that the abundant species tracked pond availability much closer than rare species. In contrast, occupied ponds for rare species were more connected than unoccupied ponds and, as a group, these species did not track pond availability as well. Spatial structure of two rare species (tiger and spotted salamanders) was reminiscent of island-mainland metapopulations (Appendix S2). These observations suggest that the rarer species were dispersal limited.

The dynamics of the chorus frog provided insight into the nature of cross-scale interactions and regime shifts that could lead to transitions between these two groups of species. The chorus frog abruptly transitioned from a rare, narrowly distributed species to an abundant, broadly distributed species, and then just as abruptly transitioned back. Fundamentally, the changes in chorus frog demography were rooted in marked temporal changes in local habitat quality and consequently landscape level availability of habitats (ponds) to this species (due to climate-driven reductions in pond predator biomass, which is positively associated with probability of extinction of chorus frogs in ESGR ponds [48]). Thus, a landscape level Moran effect (multiyear drought) altered local species interactions enabling marked growth of chorus frog populations in ponds. This scenario is consistent with niche-based explanations for generating the d-a relationship assuming no dispersal limitation (the habitat-filling model), and can generate a correlation between local density and distribution (see [16], [19]).

However, while the drought resulted in widespread changes in habitat quality and number of ponds available to the chorus frog, this alone was not sufficient to generate a correlation between density and distribution in our system. We estimated that ponds potentially suitable for the chorus frog outnumbered those actually occupied ponds by about 3.2 fold before the drought, and increased to at least 4.8-fold after initiation of the drought, suggesting strong dispersal limitation. Enhanced pond drying did lead to large increases in larval density of chorus frogs in the few ponds occupied, but the regime shift in regional population structure (15 new ponds colonized and regional population sizes 25-fold larger than the 1996 to 1999 average) only occurred after average density exceeded threshold values. This regime shift then reversed when the drought abated; declining average local density again crossed thresholds and spatial population structure subsequently collapsed. In both expansion and contraction phases, changes in local densities in occupied ponds presaged changes in the distribution of the chorus frog in space, and these density changes were clearly interpretable in terms of the mechanisms identified in experimental work [27], [47], [50].

Thus, there were strong lags in response to changes in pond availability. The inception of the drought occurred in 1998, the density shift (2000) lagged by two years, and the occupancy shift (2001) lagged by three years (the literature and analyses of population growth after colonization episodes indicate that chorus frogs can mature in one year, so these lags are not simply due to maturation time from juveniles to adults [48]). It is possible that Allee effects or lack of attraction of females to small choruses limited initial expansion of the chorus frog before local densities increased (the probability of detecting a larval population of chorus frogs given a chorus of males that spring was 0.41 if a chorus was <10 males, but 0.83 if a chorus was >10 males). Alternatively, it is possible that dispersal from a pond is strongly density-dependent. The lag extended after the drought broke as well; the drought broke in 2007 and the collapse in pond occupancy occurred in 2010. Half of the occupied ponds after the collapse were not the same ponds as those occupied before pond occupancy expansion (1996 to 2000).

Thus, both local (niche) and regional or spatial (metapopulation, but see also [55], [56]) mechanisms underlie the cross-scale interactions and regime shifts, which apparently drive macroecological patterns in this system. This system exhibits many of the characteristics conducive to such regime shifts [22], [23], e.g. nonlinear dynamics (local population growth), positive feedbacks (rescue/mass effects to occupancy), thresholds (density thresholds) and cross-scale interactions (local/regional processes). Further, a primary effect of the drought was to cause ponds on the ESGR to be more homogeneous from the perspective of the chorus frog and more connected (many more of the ponds were available). Reduced heterogeneity of components (ponds) and increased connectivity of these components also enhances the likelihood of regime shifts [23].

After the initial expansion phase, additional cross-scale interactions were important, e.g. there was a positive feedback from high local densities to overall occupancy rate as mass effects enabled presences in numerous habitats which we estimate to be sinks (closed-canopy ponds, [48]). During the 2001 to 2006 period, we estimated that 21.2±2.1% of the ponds occupied by chorus frogs were sink habitats [48]. Occupancy was erratic and densities/population sizes were always small in these habitats, as expected if they were maintained by mass/rescue effects. Thus, the realized niche of the chorus frog during this period was larger than its fundamental niche, i.e. it was consistently found in habitats arrayed along niche dimensions where birth rates were typically less than death rates. These cross-scale interactions are not typically incorporated in the mechanisms proposed for the d-a relationship.

The influence of landscape characteristics on such cross-scale interactions is signaled in differences in threshold densities on the east and west sides of the ESGR. The threshold differences were correlated with differences in mean connectivity of ponds on the two sides as assessed by circuit theory, e.g., the density threshold for the chorus frog was >2-fold higher on the east than west side associated with the >20% higher landscape resistance on the former (Appendix S2). In fact, the mean local density in occupied east ponds before expansion was higher than the threshold transition density for expansion on the west (and expansion did not occur despite a substantial number of suitable ponds available on the east side at that time). Moreover, in 2001 colonization on the west side was explosive (from 3 to 13 ponds, the maximum ever occupied), whereas on the east side pond occupancy rose from 3 to 8 ponds in 2001 but continued to gradually rise to a maximum of 13 ponds in 2009. This difference again is consistent with higher variation in mean resistance among ponds on the east side (Appendix S2). The collapse phase also indicated relevance of the thresholds, and there was a lag in the transition to the new state, i.e. local densities, regional population size and number of suitable habitats decreased while pond occupancy rates remained high before the collapse (Figure 5). Precise thresholds for regime shifts clearly will differ among species with different dispersal capabilities and differ with landscape characteristics.

We suggest that the above mechanisms are largely responsible for the interspecific d-a relationship. Niche constraints are one of the main hypothesized causes of this relationship [15], although very infrequently documented. It is likely that if these were accounted for, d-a relationships would typically appear as in Figure 2. That is, a series of species clustered on the flat portion of the curve that contribute to the upper end of the untransformed d-a curve due to differences in breadth of niche, and a group clustered lower on the curve that are more dispersal limited. Our estimates of habitats putatively available to species likely reflect these niche constraints, but are not estimated independent of the occupancy patterns reported on the same landscape. However, the patterns are consistent with what we know of the natural history of the species involved.

Species on the flat portion of the constrained d-a relationship averaged just over 60% pond occupancy, despite the fact that potential available ponds accounted for historical presences and adequate annual breeding conditions. A number of factors may contribute to suppressing the conditional occupancy below 1; e.g., predator densities vary enormously from year to year within a given pond (e.g., [48]), some fraction of the ponds inhabited may be sinks where occupancy is more stochastic, small population sizes can lead to frequent extinction events [26], [48], and detection probabilities may contribute if the species are rare in some of these ponds.

As often noted, existence of a d-a relationship suggests a “double jeopardy” where decline in one variable indicates the other likely will follow [49], [57], [58]. Thus, understanding the factors involved clearly has important implications to conservation biology, especially issues attending habitat fragmentation and critical thresholds (reviewed by Swift and Hannon [59], e.g. defined by With and King [60], as “an abrupt, nonlinear change that occurs in some parameter across a small range of habitat loss”). Factors leading to such thresholds, e.g. the interaction between habitat loss and fragmentation, Allee effects, and time lags were potentially involved in the chorus frog population dynamics. In fact, the drought essentially provided a natural experiment reversing habitat fragmentation by changing the fraction of suitable ponds on the landscape. Our results suggest that critical thresholds will be highly dependent on species and landscape characteristics (e.g., the comparison of east and west sides). However, it is clear that the change in fraction of suitable habitats alone was not sufficient to predict an expansion or collapse of the landscape level (regional) population of chorus frogs; consideration of local dynamics and the cross-scale interactions of local and regional processes were essential. The critical issue here is the scaling between species' local densities and their dispersal capacities with respect to the spatial dispersion of ponds. If we do the thought experiment of uniformly expanding distances between ESGR ponds, we would expect that species would differentially drop off the flat portion of the constrained d-a curve, and these deletions would not be orderly in regard to current species' ranks. For example, the spring peeper and the wood frog exhibited the highest average densities, but we would expect the spring peeper to drop off the flat portion much more quickly than wood frogs because the latter is capable of greater dispersal distances [61].

In conclusion, although macroecological patterns such as the d-a relationship spanning large taxonomic, spatial or temporal scales suggest that common properties underlie the structure of ecological assemblages, it has been very difficult to identify causal factors, and experiments at the requisite scales are generally perceived to be out of the question (but see [62]). Theoretical work on niche and metapopulation based processes has shown that either set of mechanisms can give rise to a correlation between distribution and abundance [9], [16], [19], but empirical work has been inconclusive. We have shown that once niche-based factors responsible for local population growth rates began to interact with processes important at larger spatial scales, this interaction enabled more complete filling of suitable habitats, which in turn fostered positive feedbacks to occupancy (maintaining sink habitats) and presumably local abundance (rescue effects). These are examples of the myriad cross-scale interactions possible in such systems, but serve to indicate the potent role they can play in determining where a species resides on the d-a curve. Moreover, large and unpredictable changes can result from cross-scale interactions of fine- with broad-scale processes (e.g., [21]) and lead to regime shifts [22], [23]. Therefore, it is not surprising that bimodal patterns in densities and occupancies are often reported (e.g., core-satellite patterns; see [7], [63]) as these patterns would be consistent with regime shifts. Studies focusing on the d-a relationship itself and attempting to draw inferences regarding which processes are responsible for it will limit progress; as with other such questions in ecology, ultimately it seems clear that what is needed is a conceptual understanding of how these processes interact to influence the d-a relationship. More mechanistic studies addressing the cross-scale interactions responsible for the relation between species abundance and distribution are critical and would inform all areas of community ecology.

Finally, our study makes clear that the d-a relationship needs to be conceptualized in a metacommunity context, which rarely has been the case [13]. By associating distribution and abundance of species, d-a relationships are essentially a bridge between local population processes and regional dynamics of species [19], [49]; this link clearly is central to understanding both metacommunity dynamics and the d-a relationship. The ESGR amphibians are part of a complex and spatially coupled metacommunity, with both the amphibians and most of their predators interacting and moving on different scales on the landscape [48], [65]. It was the climate (drought) conditioned interaction of chorus frogs and a large number of its predator species (odonate larvae, dytiscid beetle larvae, salamanders, fish, etc.), all of which are moving differentially in space in response to drought effects as well [65], that is driving the results that we have obtained. The four metacommunity models laid out by Leibold et al. [64] emphasize processes at different spatial and temporal scales, or assign different weights to the interaction of local and regional processes, and it is acknowledged that reality lies at some intersection of these models. Thus, cross-scale interactions will likely be fundamental to understanding the link between local population processes and regional dynamics of species and examining these will help integrate the conceptual frameworks associated with the d-a relationship and metacommunity ecology and provide insight into both.

## Supporting Information

Appendix S1 **Cumulative number of ponds occupied by species.**
(DOCX)

Appendix S2 **Connectivity analyses.**
(DOC)

Appendix S3 **Intraspecific distribution-abundance curves and predator biomass.**
(DOCX)

Figure S1 **Cumulative ponds occupied.** Curves of the cumulative number of ponds in which each amphibian species was sampled over the 15-yr monitoring period on the E. S. George Reserve. Species represented are: *Hyla versicolor* (Hve), *Pseudacris crucifer* (Pcr), *P. triseriata* (Ptr), *Rana catesbeiana* (Rca), *R. clamitans* (Rcl), *R. pipiens* (Rpi), *R. sylvatica* (Rsy), *Ambystoma laterale* (Ala), *A. maculatum* (Ama), *A. tigrinum* (Ati), and *Notophthalmus viridescens* (Nvi).
(TIF)

Figure S2 **Resistance landscape for the ESGR.** Warmer colors indicate paths of least resistance for amphibians. Stars represent the position of *Pseudacris triseriata* ponds on the landscape and the heavy black line divides the east and west sides of the ESGR. Friction values employed for this realization were 1, 3, 10, and 100 for wetland, forest, open and slope respectively.
(TIF)

Figure S3 **Intraspecific distribution-abundance plots for six of the eight species of ESGR amphibians.** Species represented are those on or near the flat portion of the constrained distribution-abundance relationship. Each data point represents a year.
(TIF)

Figure S4 **Dry weight biomass (± se) of predators in ponds inhabited by** ***Pseudacris triseriata*** **.** Data presented as a function of pond drying pattern where WW = wet both the previous fall and the following spring; DW = dry in the fall and wet the following spring; DD = dry in fall and dry in the following spring before filling.
(TIF)

Figure S5 **Regional population size and ponds occupied for** ***Pseudacris triseriata*** **across years versus average dry weight biomass of predators.** Top panel is regional larval population size (histograms) and mean predator biomass in ponds (line). Bottom panel is number of ponds occupied (histograms) and mean predator biomass (line).
(TIF)
