# Supplementary material for: Cross-Scale Interactions and the Distribution-Abundance Relationship
Source: PLoS One. 2014 May 29;9(5):e97387. doi: 10.1371/journal.pone.0097387 (PMC4038483; doi:10.1371/journal.pone.0097387)
Supplement: Appendix S1 — Cumulative number of ponds occupied by species. (DOCX) [file pone.0097387.s001.docx]

**Appendix S1. Cumulative number of ponds occupied by species.**

The two figure panels provided in Figure S1 present curves of the cumulative number of ponds in which each amphibian species was sampled over the 15-yr monitoring period on the E. S. George Reserve. Species represented are: *Hyla versicolor* (Hve), *Pseudacris crucifer* (Pcr), *P. triseriata* (Ptr), *Rana catesbeiana* (Rca), *R. clamitans* (Rcl), *R. pipiens* (Rpi), *R. sylvatica* (Rsy), *Ambystoma laterale* (Ala), *A. maculatum* (Ama), *A. tigrinum* (Ati), and *Notophthalmus viridescens* (Nvi). *Hemidactylum scutatum* and *R. palustris* are not presented because too few were sampled to consider for analyses. For most species cumulative curves reached an asymptote after 3 to 5 yrs of sampling. Several species exhibited more gradual increases in cumulative number of ponds occupied (e.g., *A. tigrinum*) as would be expected for species with metapopulation or island-mainland metapopulation structures. Several species also exhibited an upturn in ponds occupied in the last several years, e.g., green frogs (Rcl) and gray tree frogs (Hve). These changes can be accounted for by invasion of the emerald ash borer (Agrilus planipennis) causing death of ash trees (*Fraxinus* sp.) that formed the canopy over several ponds (both green frogs and gray tree frogs breed in open-canopy ponds and invaded these ponds after canopy removal) and, in the case of green frogs, a wetter period extending hydroperiod of some ponds. The chorus frog (Ptr) exhibited a dramatic increase in the number of ponds occupied after 2000.
