# Supplementary material for: Cross-Scale Interactions and the Distribution-Abundance Relationship
Source: PLoS One. 2014 May 29;9(5):e97387. doi: 10.1371/journal.pone.0097387 (PMC4038483; doi:10.1371/journal.pone.0097387)
Supplement: Appendix S2 — Connectivity analyses. (DOC) [file pone.0097387.s002.doc]

**Appendix S2. Connectivity analyses.**

Pond connectivity was assessed employing a resistance metric based on electric circuit theory [1] and the Hanski index (reviewed in [2,3]). The former metric provides a distance weighted according to the permeability of the landscape separating ponds and accounts for multiple potential routes between ponds; the latter takes into account distances to all other ponds containing that species and their respective population sizes. Resistance was calculated using CIRCUITSCAPE v 3.5 [1,4] from 30-m resolution composite friction grids created in ArcGIS v 9.3 [5]. Composite friction grids were generated by coding each pixel of the map as a cost to dispersal based on the type of landscape that it encompassed, with a cost of one assigned to the most permeable habitats and higher values representing less permeable habitats. This method results in correspondingly greater resistance between ponds due to landscape features incurring higher costs to movement.

Composite friction grids of the ESGR landscape (118 x 95 pixels, 3.5 x 2.9km) were composed of two components: land cover and slope. Slope was accorded an additional resistance as suggested by genetic analyses of amphibian species [6,7,8,9,10,11]. Estimates of the best fit weights for slope in genetic analyses indicate resistances of 100 to 200 times that of the habitat deemed easiest to traverse (but see [12]). Genetic analyses do not translate in any easy manner to individual movement probabilities, but these analyses qualitatively indicate that slope is a very important barrier to amphibian movement. Land cover friction grids digitized from (ground-truthed) aerial photographs included three land cover types: wetlands, forest and open areas (e.g., old fields). Wetlands were judged to be the most permeable landscape connecting ponds, and assigned a cost of one, whereas a range of cost values were examined for the other, less permeable, habitat types. Slope was calculated based on a 30-m resolution digital elevation model (Michigan Department of Natural Resources) using the slope function in the ArcGIS data management toolbox, and modeled as a linear function with a cost of zero assigned to a slope of zero and a maximum cost assigned to the highest slope possible. Slope and land cover grids were combined by summing them in ArcGIS.

Because we lack specific empirical data on the relative resistance values for different habitat types, we used the literature and our knowledge of amphibians to rank habitat types. Based on cover, vulnerability to desiccation and predation, and studies estimating the propensity to move through or into different habitats or habitat choice experiments (e.g. [13,14,15,16]), we judged that habitats would be ranked by permeability from most to least as wetlands > forest > open > slope. Our best estimate of a quantitative ranking for friction values for wetlands, forest, open and slope respectively is on the order of 1, 3, 10, and 100. Unless otherwise stated these were the values employed in analyses presented in the text. A realization of this resistance landscape is presented in Figure S2 with reference to *Pseudacris triseriata ponds* and subdivision of the ESGR discussed in the text.

To gain insight on the consequences of our choice of friction values we conducted sensitivity analyses by varying the values chosen. Since wetlands are clearly the most permeable habitat for the juvenile and adult amphibians we held resistance of this habitat constant (at a value of 1) and altered the values of the remaining habitat types. We altered forest over a range from 1 to 30 (1, 3, 5, 10, 30), open areas from 1 to 90 (1, 3, 10, 30, 90) and slope from 0 to 100 (0, 5, 10, 50, 100). Two very large ponds with fish were given high values (100) as they would be dangerous for small frogs to cross. Results of the sensitivity analyses are presented in Table 1.

We also employed the Hanski connectivity index for pond *i*, *S_i_*, taking into account distances to all other ponds on the ESGR containing that species and their respective population sizes (reviewed in [2,3]):

$$S_{i}=\sum_{j\neq i} \exp\left(-\alpha d_{ij}\right)N_{j}$$

where α scales the effect of distance to dispersal (1/α is the mean dispersal distance), *d_ij_* is the distance (edge to edge) between ponds *i* and *j*, and *N_j_* is the larval population size of the species in pond *j* (thus we assume larval population size is correlated with numbers of dispersing individuals). This index therefore accounts for spatial position and population densities of ponds but is independent of intervening terrestrial habitat characteristics. To employ this index, we estimated the time to reproductive maturity for the species (which may be a range as all individuals do not mature synchronously) in question and average dispersal distance from the literature. Time to first reproduction is required because we need to know when our measure of population density, the larval population in pond *j*, potentially influences the breeding adult population of pond *i* and average dispersal distance enables an estimate of α. If time to maturity was a range, we used the average of larval populations over the designated preceding years. We employed the following values for each species (mean dispersal distance and time to reproduction respectively): *Ambystoma tigrinum* (125 m, 2 to 4 yrs), *A. maculatum* (125 m, 2 to 3 yrs), *A. laterale* (125 m, 2 to 3 yrs), *Rana pipiens* (200 m, 2 to 3 yrs), *R. sylvatica* (200 m, 2 to 3 yrs), *Pseudacris triseriata* (100 m, 1 to 2 yrs), and *P. crucifer* (100 m, 1 to 2 yrs). Time to reproduction and dispersal distances were based on our experience and previous estimates [17,18,19,20].

The spotted and tiger salamanders exhibited population structures suggestive of island/mainland metapopulations. For example, the tiger salamander was sampled in 87% of years in one pond and an average of 67% of years in three additional ponds; most other ponds exhibited very sporadic presences (14 ponds with presences averaging 23% of years). Similarly, the spotted salamander was consistently found in one pond (87% occupancy rate) and three others with an average frequency of 55%. No other pond had > 27% occupancy rate and these ponds (n = 12) averaged 16% occupancy. Ponds that were intermittently colonized from those we identified as source ponds were more connected to those sources than those that were not colonized for both species (e.g., comparing cumulative “current” to colonized ponds compared to non colonized ponds; tiger salamander with one outlier pond, Grubbs test = 3.45 with 5% cutoff = 2.53, removed from the analysis, t-test, t (30) = 2.84, p = 0.004, spotted salamander, t-test, t (50) = 2.1, p = 0.049). Employing the Hanski index, newly colonized ponds exhibited significantly higher connectivity than those not colonized for the tiger salamander (t-test, ln-transformed data, t (396) = 4.1, p < 0.001) whereas for the spotted salamander newly colonized ponds exhibited a trend to higher connectivity (1.5 fold higher), but the difference was not significant.

**Literature Cited**

1. McRae BH (2006) Isolation by resistance. Evolution 60: 1551-1561.

2. Moilanen A, Nieminen M (2002) Simple connectivity measures in spatial ecology. Ecology 83: 1131-1145.

3. Winfree R, Dushoff J, Crone EE, Schultz CB, Budny RV, et al. (2005) Testing simple indices of habitat proximity. American Naturalist 165: 707-717.

4. McRae BH, Dickson BG, Keitt TH, Shah VB (2008) Using circuit theory to model connectivity in ecology, evolution, and conservation. Ecology 89: 2712-2724.

5. ESRI (2008) ArcGIS Desktop: Version 9.3. Redlands, CA: Environmental Systems Research Institute.

6. Funk WC, Blouin MS, Corn PS, Maxell BA, Pilliod DS, et al. (2005) Population structure of Columbia spotted frogs (*Rana luteiventris*) is strongly affected by the landscape. Molecular Ecology 14: 483-496.

7. Giordano AR, Ridenhour BJ, Storfer A (2007) The influence of altitude and topography on genetic structure in the long-toed salamander (*Ambystoma macrodactulym*). Molecular Ecology 16: 1625-1637.

8. Lowe WH, Likens GE, McPeek MA, Buso DC (2006) Linking direct and indirect data on dispersal: Isolation by slope in a headwater stream salamander. Ecology 87: 334-339.

9. Richards-Zawacki CL (2009) Effects of slope and riparian habitat connectivity on gene flow in an endangered Panamanian frog, *Atelopus varius*. Diversity and Distributions 15: 796-806.

10. Spear SF, Storfer A (2010) Anthropogenic and natural disturbance lead to differing patterns of gene flow in the Rocky Mountain tailed frog, *Ascaphus montanus*. Biological Conservation 143: 778-786.

11. Zellmer AJ, Knowles LL (2009) Disentangling the effects of historic vs. contemporary landscape structure on population genetic divergence. Molecular Ecology 18: 3593-3602.

12. Boone RB, Johnson CM, Johnson LB (2006) Simulating wood frog movement in central Minnesota, USA using a diffusion model. Ecological Modelling 198: 255-262.

13. Gibbs JP (1998) Amphibian movements in response to forest edges, roads, and streambeds in southern New England. Journal of Wildlife Management 62: 584-589.

14. Popescu VD, Hunter ML, Jr. (2011) Clear-cutting affects habitat connectivity for a forest amphibian by decreasing permeability to juvenile movements. Ecological Applications 21: 1283-1295.

15. Rothermel BB, Semlitsch RD (2002) An experimental investigation of landscape resistance of forest versus old-field habitats to emigrating juvenile amphibians. Conservation Biology 16: 1324-1332.

16. Vos CC, Goedhart PW, Lammertsma DR, Spitzen-Van der Sluijs AM (2007) Matrix permeability of agricultural landscapes: an analysis of movements of the common frog (*Rana temporaria*). Herpetological Journal 17: 174-182.

17. Lannoo MJ, editor (2005) Amphibian Declines: The Conservation Status of U.S. Amphibians. Berkeley, California, USA: University of California Press. 1094 p.

18. Semlitsch RD (1998) Biological delineation of terrestrial buffer zones for pond-breeding salamanders. Conservation Biology 12: 1113-1119.

19. Smith MA, Green DM (2005) Dispersal and the metapopulation paradigm in amphibian ecology and conservation: are all amphibian populations metapopulations? Ecography 28: 110-128.

20. Wright AH, Wright AA (1949) Handbook of frogs and toads of the United States and Canada. Ithaca, New York, USA: Comstock.

**Table S2.1.** Sensitivity Analyses^*^.

| Wetland | Forest | Open | Slope |  | Global mean resistance | East mean resistance | West mean resistance | East:West mean resistance |  | Global variance | East variance | West variance | East:West variance |
| --- | --- | --- | --- | --- | --- | --- | --- | --- | --- | --- | --- | --- | --- |
| 1 | 1 | 1 | 0 |  | 0.64 | 0.5 | 0.53 | 0.93 |  | 0.032 | 0.016 | 0.027 | 0.596 |
| 1 | 1 | 1 | 5 |  | 1.25 | 1.06 | 0.98 | 1.09 |  | 0.117 | 0.075 | 0.084 | 0.895 |
| 1 | 1 | 1 | 10 |  | 1.81 | 1.59 | 1.39 | 1.14 |  | 0.251 | 0.181 | 0.167 | 1.083 |
| 1 | 1 | 1 | 50 |  | 5.99 | 5.6 | 4.48 | 1.25 |  | 3.026 | 2.751 | 1.751 | 1.571 |
| 1 | 1 | 1 | 100 |  | 11.1 | 10.53 | 8.29 | 1.27 |  | 10.732 | 10.309 | 6.001 | 1.718 |
| 1 | 1 | 3 | 0 |  | 0.71 | 0.54 | 0.58 | 0.93 |  | 0.044 | 0.02 | 0.037 | 0.539 |
| 1 | 1 | 3 | 5 |  | 1.35 | 1.12 | 1.05 | 1.07 |  | 0.147 | 0.082 | 0.111 | 0.741 |
| 1 | 1 | 3 | 10 |  | 1.92 | 1.66 | 1.46 | 1.13 |  | 0.298 | 0.191 | 0.211 | 0.907 |
| 1 | 1 | 3 | 50 |  | 6.15 | 5.69 | 4.59 | 1.24 |  | 3.223 | 2.79 | 1.935 | 1.441 |
| 1 | 1 | 3 | 100 |  | 11.28 | 10.63 | 8.4 | 1.27 |  | 11.126 | 10.389 | 6.368 | 1.631 |
| 1 | 1 | 5 | 0 |  | 0.74 | 0.56 | 0.6 | 0.93 |  | 0.052 | 0.024 | 0.043 | 0.551 |
| 1 | 1 | 5 | 5 |  | 1.4 | 1.16 | 1.08 | 1.07 |  | 0.168 | 0.089 | 0.129 | 0.687 |
| 1 | 1 | 5 | 10 |  | 1.99 | 1.7 | 1.51 | 1.12 |  | 0.331 | 0.2 | 0.242 | 0.828 |
| 1 | 1 | 5 | 50 |  | 6.27 | 5.77 | 4.67 | 1.23 |  | 3.377 | 2.825 | 2.087 | 1.353 |
| 1 | 1 | 5 | 100 |  | 11.43 | 10.72 | 8.5 | 1.26 |  | 11.456 | 10.457 | 6.688 | 1.564 |
| 1 | 1 | 10 | 0 |  | 0.79 | 0.59 | 0.63 | 0.94 |  | 0.063 | 0.031 | 0.051 | 0.601 |
| 1 | 1 | 10 | 5 |  | 1.49 | 1.21 | 1.13 | 1.07 |  | 0.201 | 0.102 | 0.158 | 0.646 |
| 1 | 1 | 10 | 10 |  | 2.1 | 1.77 | 1.58 | 1.12 |  | 0.389 | 0.219 | 0.294 | 0.745 |
| 1 | 1 | 10 | 50 |  | 6.49 | 5.9 | 4.82 | 1.22 |  | 3.682 | 2.892 | 2.395 | 1.207 |
| 1 | 1 | 10 | 100 |  | 11.71 | 10.9 | 8.7 | 1.25 |  | 12.13 | 10.593 | 7.365 | 1.438 |
| 1 | 1 | 30 | 0 |  | 0.84 | 0.63 | 0.66 | 0.95 |  | 0.081 | 0.044 | 0.063 | 0.704 |
| 1 | 1 | 30 | 5 |  | 1.61 | 1.29 | 1.21 | 1.07 |  | 0.263 | 0.133 | 0.206 | 0.645 |
| 1 | 1 | 30 | 10 |  | 2.28 | 1.89 | 1.69 | 1.12 |  | 0.506 | 0.27 | 0.392 | 0.69 |
| 1 | 1 | 30 | 50 |  | 6.15 | 5.69 | 4.59 | 1.24 |  | 3.223 | 2.79 | 1.935 | 1.441 |
| 1 | 1 | 30 | 100 |  | 12.41 | 11.34 | 9.18 | 1.24 |  | 13.962 | 11.047 | 9.253 | 1.194 |
| 1 | 3 | 1 | 0 |  | 1.37 | 1.07 | 1.21 | 0.88 |  | 0.172 | 0.097 | 0.175 | 0.552 |
| 1 | 3 | 1 | 5 |  | 1.97 | 1.63 | 1.66 | 0.98 |  | 0.321 | 0.219 | 0.284 | 0.772 |
| 1 | 3 | 1 | 10 |  | 2.54 | 2.17 | 2.08 | 1.04 |  | 0.517 | 0.389 | 0.419 | 0.93 |
| 1 | 3 | 1 | 50 |  | 6.76 | 6.21 | 5.22 | 1.19 |  | 3.797 | 3.473 | 2.405 | 1.444 |
| 1 | 3 | 1 | 100 |  | 11.9 | 11.15 | 9.04 | 1.23 |  | 12.137 | 11.684 | 7.159 | 1.632 |
| 1 | 3 | 3 | 0 |  | 1.5 | 1.15 | 1.31 | 0.88 |  | 0.219 | 0.11 | 0.226 | 0.487 |
| 1 | 3 | 3 | 5 |  | 2.12 | 1.72 | 1.76 | 0.98 |  | 0.388 | 0.236 | 0.354 | 0.667 |
| 1 | 3 | 3 | 10 |  | 2.69 | 2.26 | 2.19 | 1.03 |  | 0.603 | 0.409 | 0.506 | 0.809 |
| 1 | 3 | 3 | 50 |  | 6.94 | 6.31 | 5.34 | 1.18 |  | 4.041 | 3.525 | 2.647 | 1.332 |
| 1 | 3 | 3 | 100 |  | 12.09 | 11.26 | 9.17 | 1.23 |  | 12.586 | 11.777 | 7.588 | 1.552 |
| 1 | 3 | 5 | 0 |  | 1.58 | 1.2 | 1.36 | 0.88 |  | 0.25 | 0.122 | 0.257 | 0.476 |
| 1 | 3 | 5 | 5 |  | 2.2 | 1.77 | 1.82 | 0.97 |  | 0.433 | 0.252 | 0.401 | 0.628 |
| 1 | 3 | 5 | 10 |  | 2.78 | 2.32 | 2.25 | 1.03 |  | 0.664 | 0.427 | 0.57 | 0.75 |
| 1 | 3 | 5 | 50 |  | 7.08 | 6.39 | 5.43 | 1.18 |  | 4.238 | 3.571 | 2.835 | 1.259 |
| 1 | 3 | 5 | 100 |  | 12.24 | 11.36 | 9.27 | 1.22 |  | 12.967 | 11.838 | 7.969 | 1.485 |
| 1 | 3 | 10 | 0 |  | 1.68 | 1.26 | 1.43 | 0.88 |  | 0.299 | 0.147 | 0.304 | 0.485 |
| 1 | 3 | 10 | 5 |  | 2.33 | 1.86 | 1.91 | 0.97 |  | 0.512 | 0.282 | 0.479 | 0.589 |
| 1 | 3 | 10 | 10 |  | 2.93 | 2.41 | 2.36 | 1.02 |  | 0.773 | 0.465 | 0.68 | 0.684 |
| 1 | 3 | 10 | 50 |  | 7.32 | 6.55 | 5.6 | 1.17 |  | 4.624 | 3.669 | 3.24 | 1.132 |
| 1 | 3 | 10 | 100 |  | 12.55 | 11.55 | 9.48 | 1.22 |  | 13.734 | 12.032 | 8.748 | 1.375 |
| 1 | 3 | 30 | 0 |  | 1.83 | 1.37 | 1.52 | 0.9 |  | 0.39 | 0.211 | 0.379 | 0.558 |
| 1 | 3 | 30 | 5 |  | 2.55 | 2.01 | 2.05 | 0.98 |  | 0.671 | 0.371 | 0.62 | 0.599 |
| 1 | 3 | 30 | 10 |  | 3.2 | 2.6 | 2.53 | 1.03 |  | 1.005 | 0.578 | 0.895 | 0.646 |
| 1 | 3 | 30 | 50 |  | 6.94 | 6.31 | 5.34 | 1.18 |  | 4.043 | 3.528 | 2.645 | 1.334 |
| 1 | 3 | 30 | 100 |  | 13.3 | 12.03 | 10.01 | 1.2 |  | 15.822 | 12.583 | 10.946 | 1.15 |
| 1 | 5 | 1 | 0 |  | 1.99 | 1.57 | 1.81 | 0.87 |  | 0.428 | 0.278 | 0.458 | 0.608 |
| 1 | 5 | 1 | 5 |  | 2.61 | 2.15 | 2.28 | 0.94 |  | 0.638 | 0.464 | 0.617 | 0.752 |
| 1 | 5 | 1 | 10 |  | 3.19 | 2.69 | 2.71 | 0.99 |  | 0.896 | 0.697 | 0.799 | 0.872 |
| 1 | 5 | 1 | 50 |  | 7.48 | 6.78 | 5.91 | 1.15 |  | 4.672 | 4.293 | 3.193 | 1.345 |
| 1 | 5 | 1 | 100 |  | 12.64 | 11.74 | 9.76 | 1.2 |  | 13.64 | 13.159 | 8.425 | 1.562 |
| 1 | 5 | 3 | 0 |  | 2.17 | 1.67 | 1.94 | 0.86 |  | 0.518 | 0.301 | 0.56 | 0.538 |
| 1 | 5 | 3 | 5 |  | 2.79 | 2.25 | 2.41 | 0.94 |  | 0.747 | 0.49 | 0.736 | 0.666 |
| 1 | 5 | 3 | 10 |  | 3.37 | 2.8 | 2.84 | 0.99 |  | 1.025 | 0.728 | 0.94 | 0.775 |
| 1 | 5 | 3 | 50 |  | 7.67 | 6.89 | 6.04 | 1.14 |  | 4.963 | 4.362 | 3.48 | 1.254 |
| 1 | 5 | 3 | 100 |  | 12.85 | 11.86 | 9.9 | 1.2 |  | 14.14 | 13.275 | 8.93 | 1.487 |
| 1 | 5 | 5 | 0 |  | 2.27 | 1.74 | 2.02 | 0.86 |  | 0.576 | 0.322 | 0.625 | 0.515 |
| 1 | 5 | 5 | 5 |  | 2.9 | 2.32 | 2.48 | 0.93 |  | 0.822 | 0.513 | 0.819 | 0.626 |
| 1 | 5 | 5 | 10 |  | 3.48 | 2.87 | 2.92 | 0.98 |  | 1.117 | 0.753 | 1.04 | 0.724 |
| 1 | 5 | 5 | 50 |  | 7.82 | 6.98 | 6.14 | 1.14 |  | 5.196 | 4.413 | 3.717 | 1.187 |
| 1 | 5 | 5 | 100 |  | 13.01 | 11.95 | 10 | 1.19 |  | 14.548 | 13.338 | 9.348 | 1.427 |
| 1 | 5 | 10 | 0 |  | 2.41 | 1.83 | 2.12 | 0.87 |  | 0.673 | 0.367 | 0.727 | 0.505 |
| 1 | 5 | 10 | 5 |  | 3.07 | 2.43 | 2.6 | 0.93 |  | 0.952 | 0.565 | 0.96 | 0.588 |
| 1 | 5 | 10 | 10 |  | 3.67 | 2.99 | 3.05 | 0.98 |  | 1.282 | 0.812 | 1.22 | 0.666 |
| 1 | 5 | 10 | 50 |  | 8.08 | 7.15 | 6.33 | 1.13 |  | 5.656 | 4.524 | 4.205 | 1.076 |
| 1 | 5 | 10 | 100 |  | 13.33 | 12.17 | 10.23 | 1.19 |  | 15.426 | 13.587 | 10.241 | 1.327 |
| 1 | 5 | 30 | 0 |  | 2.65 | 2 | 2.27 | 0.88 |  | 0.864 | 0.493 | 0.905 | 0.545 |
| 1 | 5 | 30 | 5 |  | 3.36 | 2.63 | 2.79 | 0.94 |  | 1.228 | 0.717 | 1.229 | 0.583 |
| 1 | 5 | 30 | 10 |  | 4.01 | 3.22 | 3.28 | 0.98 |  | 1.643 | 0.99 | 1.58 | 0.627 |
| 1 | 5 | 30 | 50 |  | 8.68 | 7.55 | 6.74 | 1.12 |  | 6.826 | 4.925 | 5.454 | 0.903 |
| 1 | 5 | 30 | 100 |  | 14.12 | 12.67 | 10.78 | 1.18 |  | 17.77 | 14.194 | 12.764 | 1.112 |
| 1 | 10 | 1 | 0 |  | 3.38 | 2.7 | 3.17 | 0.85 |  | 1.615 | 1.22 | 1.775 | 0.687 |
| 1 | 10 | 1 | 5 |  | 4.05 | 3.32 | 3.69 | 0.9 |  | 1.967 | 1.549 | 2.046 | 0.757 |
| 1 | 10 | 1 | 10 |  | 4.67 | 3.89 | 4.16 | 0.93 |  | 2.371 | 1.936 | 2.349 | 0.824 |
| 1 | 10 | 1 | 50 |  | 9.13 | 8.11 | 7.52 | 1.08 |  | 7.34 | 6.796 | 5.73 | 1.186 |
| 1 | 10 | 1 | 100 |  | 14.39 | 13.15 | 11.46 | 1.15 |  | 17.83 | 17.271 | 12.219 | 1.413 |
| 1 | 10 | 3 | 0 |  | 3.64 | 2.86 | 3.38 | 0.85 |  | 1.827 | 1.27 | 2.029 | 0.626 |
| 1 | 10 | 3 | 5 |  | 4.3 | 3.46 | 3.88 | 0.89 |  | 2.195 | 1.604 | 2.314 | 0.693 |
| 1 | 10 | 3 | 10 |  | 4.91 | 4.03 | 4.34 | 0.93 |  | 2.619 | 1.996 | 2.634 | 0.758 |
| 1 | 10 | 3 | 50 |  | 9.36 | 8.25 | 7.67 | 1.08 |  | 7.784 | 6.931 | 6.15 | 1.127 |
| 1 | 10 | 3 | 100 |  | 14.62 | 13.29 | 11.61 | 1.14 |  | 18.47 | 17.417 | 12.87 | 1.353 |
| 1 | 10 | 5 | 0 |  | 3.79 | 2.95 | 3.49 | 0.84 |  | 1.968 | 1.311 | 2.205 | 0.595 |
| 1 | 10 | 5 | 5 |  | 4.45 | 3.56 | 3.99 | 0.89 |  | 2.355 | 1.649 | 2.507 | 0.658 |
| 1 | 10 | 5 | 10 |  | 5.07 | 4.13 | 4.45 | 0.93 |  | 2.798 | 2.044 | 2.848 | 0.718 |
| 1 | 10 | 5 | 50 |  | 9.52 | 8.35 | 7.79 | 1.07 |  | 8.084 | 6.98 | 6.496 | 1.074 |
| 1 | 10 | 5 | 100 |  | 14.8 | 13.4 | 11.73 | 1.14 |  | 19.016 | 17.57 | 13.4 | 1.311 |
| 1 | 10 | 10 | 0 |  | 4.02 | 3.1 | 3.66 | 0.85 |  | 2.208 | 1.404 | 2.497 | 0.562 |
| 1 | 10 | 10 | 5 |  | 4.7 | 3.72 | 4.17 | 0.89 |  | 2.638 | 1.747 | 2.85 | 0.613 |
| 1 | 10 | 10 | 10 |  | 5.32 | 4.29 | 4.64 | 0.93 |  | 3.123 | 2.148 | 3.235 | 0.664 |
| 1 | 10 | 10 | 50 |  | 9.84 | 8.55 | 8.02 | 1.07 |  | 8.748 | 7.162 | 7.266 | 0.986 |
| 1 | 10 | 10 | 100 |  | 15.16 | 13.62 | 11.99 | 1.14 |  | 20.115 | 17.804 | 14.601 | 1.219 |
| 1 | 10 | 30 | 0 |  | 4.43 | 3.39 | 3.94 | 0.86 |  | 2.722 | 1.704 | 3.064 | 0.556 |
| 1 | 10 | 30 | 5 |  | 5.15 | 4.03 | 4.48 | 0.9 |  | 3.267 | 2.073 | 3.553 | 0.583 |
| 1 | 10 | 30 | 10 |  | 5.82 | 4.64 | 4.98 | 0.93 |  | 3.864 | 2.499 | 4.07 | 0.614 |
| 1 | 10 | 30 | 50 |  | 10.56 | 9.02 | 8.53 | 1.06 |  | 10.441 | 7.7 | 9.158 | 0.841 |
| 1 | 10 | 30 | 100 |  | 16.05 | 14.19 | 12.62 | 1.12 |  | 23.122 | 18.69 | 17.918 | 1.043 |
| 1 | 30 | 1 | 0 |  | 8.3 | 6.82 | 8.17 | 0.84 |  | 14.674 | 12.358 | 16.099 | 0.768 |
| 1 | 30 | 1 | 5 |  | 9.15 | 7.55 | 8.85 | 0.85 |  | 15.43 | 13.161 | 16.676 | 0.789 |
| 1 | 30 | 1 | 10 |  | 9.9 | 8.21 | 9.43 | 0.87 |  | 16.312 | 14.025 | 17.352 | 0.808 |
| 1 | 30 | 1 | 50 |  | 14.95 | 12.88 | 13.3 | 0.97 |  | 25.608 | 23.621 | 24.287 | 0.973 |
| 1 | 30 | 1 | 100 |  | 20.61 | 18.22 | 17.59 | 1.04 |  | 41.946 | 40.375 | 35.617 | 1.134 |
| 1 | 30 | 3 | 0 |  | 8.79 | 7.09 | 8.55 | 0.83 |  | 15.378 | 12.546 | 17.028 | 0.737 |
| 1 | 30 | 3 | 5 |  | 9.58 | 7.79 | 9.17 | 0.85 |  | 16.17 | 13.295 | 17.629 | 0.754 |
| 1 | 30 | 3 | 10 |  | 10.3 | 8.44 | 9.73 | 0.87 |  | 17.08 | 14.224 | 18.344 | 0.775 |
| 1 | 30 | 3 | 50 |  | 15.28 | 13.07 | 13.52 | 0.97 |  | 26.596 | 23.903 | 25.417 | 0.94 |
| 1 | 30 | 3 | 100 |  | 20.91 | 18.39 | 17.79 | 1.03 |  | 43.152 | 40.639 | 36.946 | 1.1 |
| 1 | 30 | 5 | 0 |  | 9.07 | 7.26 | 8.79 | 0.83 |  | 15.949 | 12.61 | 17.854 | 0.706 |
| 1 | 30 | 5 | 5 |  | 9.85 | 7.95 | 9.39 | 0.85 |  | 16.772 | 13.429 | 18.456 | 0.728 |
| 1 | 30 | 5 | 10 |  | 10.56 | 8.6 | 9.93 | 0.87 |  | 17.7 | 14.349 | 19.183 | 0.748 |
| 1 | 30 | 5 | 50 |  | 15.52 | 13.22 | 13.7 | 0.96 |  | 27.423 | 24.155 | 26.4 | 0.915 |
| 1 | 30 | 5 | 100 |  | 21.15 | 18.54 | 17.96 | 1.03 |  | 44.155 | 40.998 | 38.039 | 1.078 |
| 1 | 30 | 10 | 0 |  | 9.52 | 7.55 | 9.15 | 0.82 |  | 16.972 | 12.895 | 19.317 | 0.668 |
| 1 | 30 | 10 | 5 |  | 10.3 | 8.24 | 9.74 | 0.85 |  | 17.857 | 13.737 | 19.969 | 0.688 |
| 1 | 30 | 10 | 10 |  | 11.02 | 8.88 | 10.29 | 0.86 |  | 18.853 | 14.629 | 20.775 | 0.704 |
| 1 | 30 | 10 | 50 |  | 15.98 | 13.5 | 14.04 | 0.96 |  | 28.972 | 24.436 | 28.361 | 0.862 |
| 1 | 30 | 10 | 100 |  | 21.62 | 18.83 | 18.3 | 1.03 |  | 46.257 | 41.494 | 40.495 | 1.025 |
| 1 | 30 | 30 | 0 |  | 10.42 | 8.17 | 9.83 | 0.83 |  | 19.39 | 13.888 | 22.692 | 0.612 |
| 1 | 30 | 30 | 5 |  | 11.22 | 8.88 | 10.44 | 0.85 |  | 20.483 | 14.729 | 23.561 | 0.625 |
| 1 | 30 | 30 | 10 |  | 11.96 | 9.53 | 11 | 0.87 |  | 21.65 | 15.715 | 24.535 | 0.641 |
| 1 | 30 | 30 | 50 |  | 17.06 | 14.22 | 14.85 | 0.96 |  | 33.056 | 25.754 | 33.61 | 0.766 |
| 1 | 30 | 30 | 100 |  | 22.82 | 19.61 | 19.19 | 1.02 |  | 52.004 | 43.119 | 47.513 | 0.908 |

^*^This table provides the mean resistance between ponds and its variance computed across all ponds on the E. S. George Reserve (global values) as well as the values for the eastern and westerns sides of the Reserve computed separately. We also provide the ratio of east:west values. These values were computed for a range of different friction values for each habitat type (other than wetlands). The first four columns indicate the specific friction values accorded each habitat type and the remaining eight columns the mean resistance values among ponds and their variances.
