# Supplementary material for: Cross-Scale Interactions and the Distribution-Abundance Relationship
Source: PLoS One. 2014 May 29;9(5):e97387. doi: 10.1371/journal.pone.0097387 (PMC4038483; doi:10.1371/journal.pone.0097387)
Supplement: Appendix S3 — Intraspecific distribution-abundance curves and predator biomass. (DOCX) [file pone.0097387.s003.docx]

**Appendix S3.** **Intraspecific distribution-abundance curves and predator biomass.**

*Intraspecific d-a curves.* Figure S3 presents d-a relationships for individual species over the 15 yrs, i.e. mean density of the species in occupied ponds each year plotted against the number of ponds occupied that year. Data are for six of the eight species of ESGR amphibians on or near the flat portion of the constrained d-a relationship. Omitted were the chorus frog (*Pseudacris triseriata)* whose dynamics are treated in detail in the body of the paper and the bullfrog (*Rana catesbieana)* which only occupied 1-3 ponds over the survey period. ). Of the eight species on the flat portion of the curve, p values for linear regressions all ranged between 0.20 and 0.50 with the exception of *A. laterale* (p = 0.08, due to lower densities and occupancies in 2000, 2003 and 2004 when spring filling of ponds was late prohibiting breeding of this very early spring breeding species) and chorus frogs. These species exhibited no relation intraspecifically between density and occupancy despite the fact that densities varied widely across years (e.g., average spring peeper densities ranged from 3 to 26/m^2^ and wood frog densities from 3 to nearly 28/m^2^ with no effect on occupancy rates (the three lower values for wood frogs again in 2000, 2003 and 2004 for reasons indicated above).

*Pond drying and predator biomass.* To illustrate the role of pond drying on predator biomass in ponds we calculated May predator biomass in a pond as a function of three conditions, i.e. whether the pond 1) held water the previous fall, 2) dried the previous fall, or 3) dried the previous fall and was dry in the spring of the current year before filling. We performed these analyses on all ponds that had ever contained a chorus frog population. We summed dry weight biomass (see [1,2] for details) of all fish, all dragonfly larvae except *Sympetrum* (a genus not dangerous to chorus frogs in the spring due to small body size), newts (*Notophthalmus viridescens*) and tiger salamanders (*Ambystoma tigrinum*), adult and larval Dytiscidae, all Hemiptera, larval Hydrophilidae, and Megaloptera.

Ponds that dried the previous fall had only 25.5% of the predator biomass found in ponds that did not dry, and ponds that were also dry in the early spring and then refilled only 14% of predator biomass found in pond that did not dry (Figure S4, ln transformed data, ANOVA, F_2, 394_ = 11.7, P < 0.001, all categories differed from each other at P values < 0.003, Tamhane test). Taxa especially affected were fish and odonate larvae; if a pond dried in the fall these taxa exhibited < 5% of the biomass of ponds that did not dry (odonates F_2, 394_ = 124.8, p << 0.001; fish F_2, 397_ = 17.9, p < 0.001). Hemiptera exhibited 41% of the biomass if the pond dried compared to ponds that remained wet (F_2, 397_ = 28.8, p < 0.001) and tiger salamanders 70% (not significant). However, most predator taxa overwintering as adults and initiating breeding in the spring exhibited little or no biomass reduction due to fall drying (e.g., Hydrophilidae, Dytiscidae, Megaloptera, and Salamandridae). Closed-canopy ponds were not differentiated with respect to drying as predator biomass was not affected because of the species present in these ponds (t-test, df = 1, 158, t = -0.95, p = 0.93).

*Chorus frog dynamics and predator biomass.* Chorus frog regional population and pond occupancy exhibited a strong association with patterns in pond drying and predator densities, but with clear lags associated with both the initial population expansion and the population crash (Figure S5).

**Literature Cited**

1. Werner EE, Relyea RA, Yurewicz KL, Skelly DK, Davis CJ (2009) Comparative landscape dynamics of two anuran species: climate-driven interaction of local and regional processes. Ecological Monographs 79: 503-521.

2. Werner EE, Skelly DK, Relyea RA, Yurewicz KL (2007) Amphibian species richness across environmental gradients. Oikos 116: 1697-1712.
