# Supplementary figures and images for: Cross-Scale Interactions and the Distribution-Abundance Relationship
Source: PLoS One. 2014 May 29;9(5):e97387. doi: 10.1371/journal.pone.0097387 (PMC4038483; doi:10.1371/journal.pone.0097387)

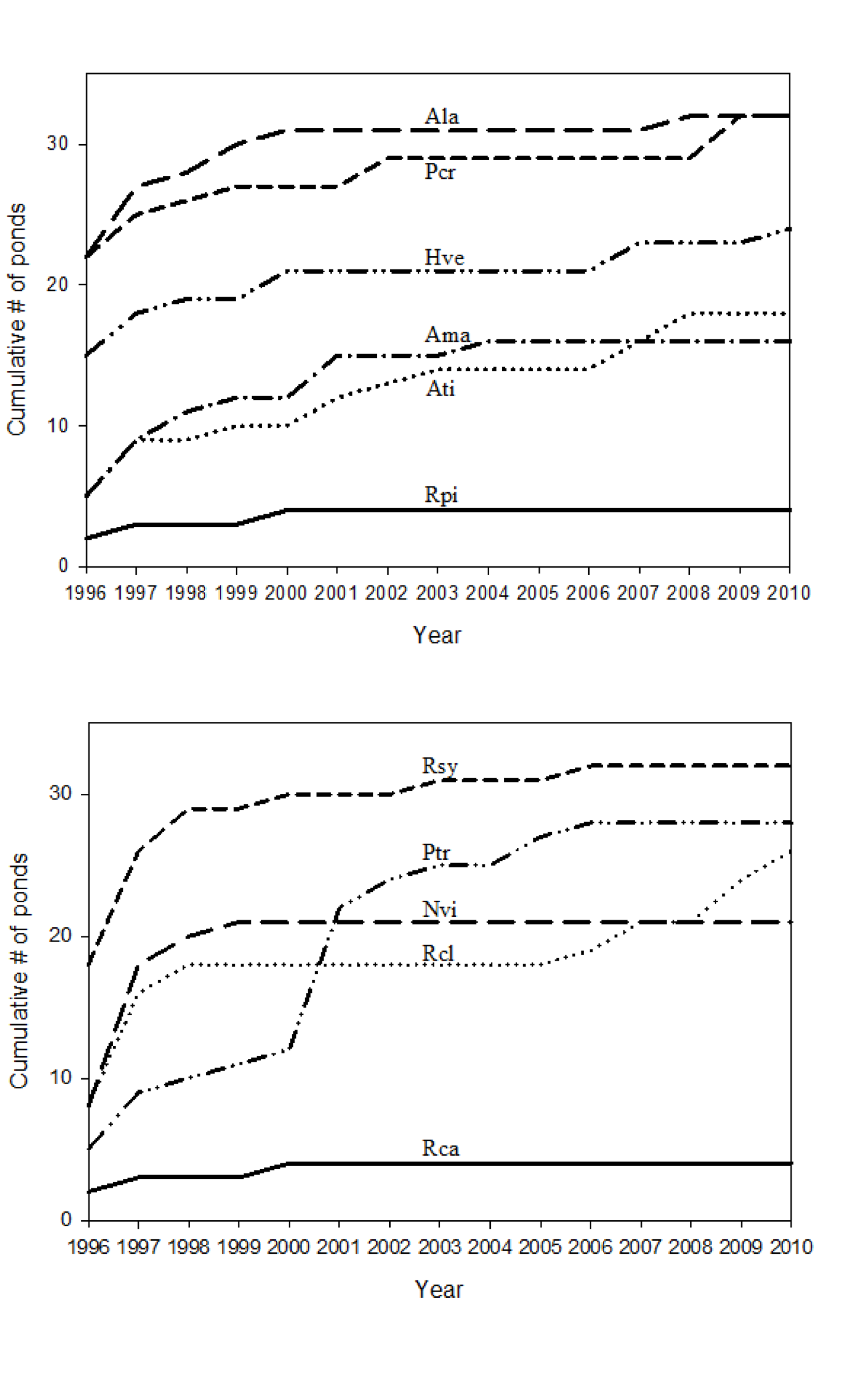

Supplement: Figure S1 — Cumulative ponds occupied. Curves of the cumulative number of ponds in which each amphibian species was sampled over the 15-yr monitoring period on the E. S. George Reserve. Species represented are: Hyla versicolor (Hve), Pseudacris crucifer (Pcr), P. triseriata (Ptr), Rana catesbeiana (Rca), R. clamitans (Rcl), R. pipiens (Rpi), R. sylvatica (Rsy), Ambystoma laterale (Ala), A. maculatum (Ama), A. tigrinum (Ati), and Notophthalmus viridescens (Nvi). (TIF) [file pone.0097387.s004.tif]

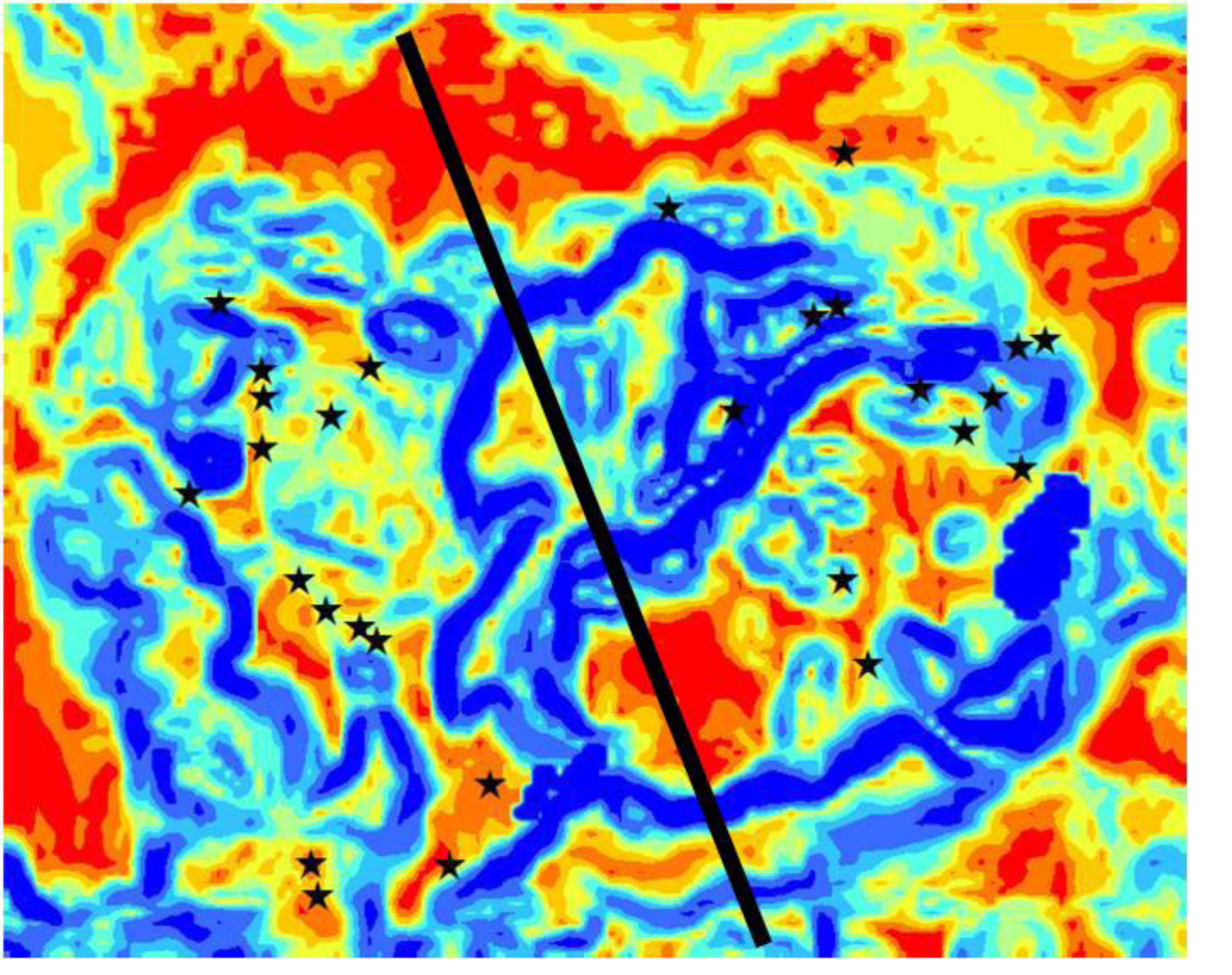

Supplement: Figure S2 — Resistance landscape for the ESGR. Warmer colors indicate paths of least resistance for amphibians. Stars represent the position of Pseudacris triseriata ponds on the landscape and the heavy black line divides the east and west sides of the ESGR. Friction values employed for this realization were 1, 3, 10, and 100 for wetland, forest, open and slope respectively. (TIF) [file pone.0097387.s005.tif]

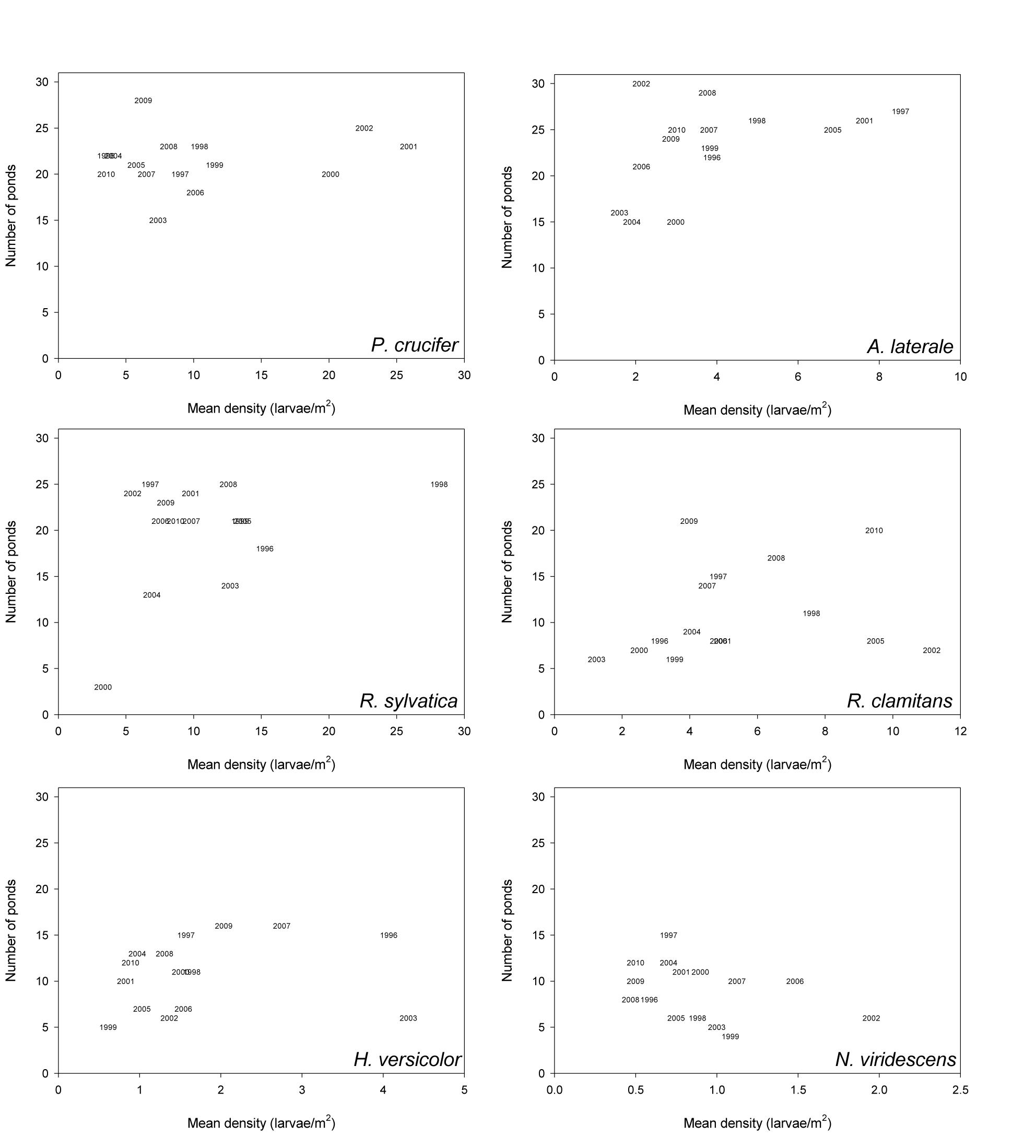

Supplement: Figure S3 — Intraspecific distribution-abundance plots for six of the eight species of ESGR amphibians. Species represented are those on or near the flat portion of the constrained distribution-abundance relationship. Each data point represents a year. (TIF) [file pone.0097387.s006.tif]

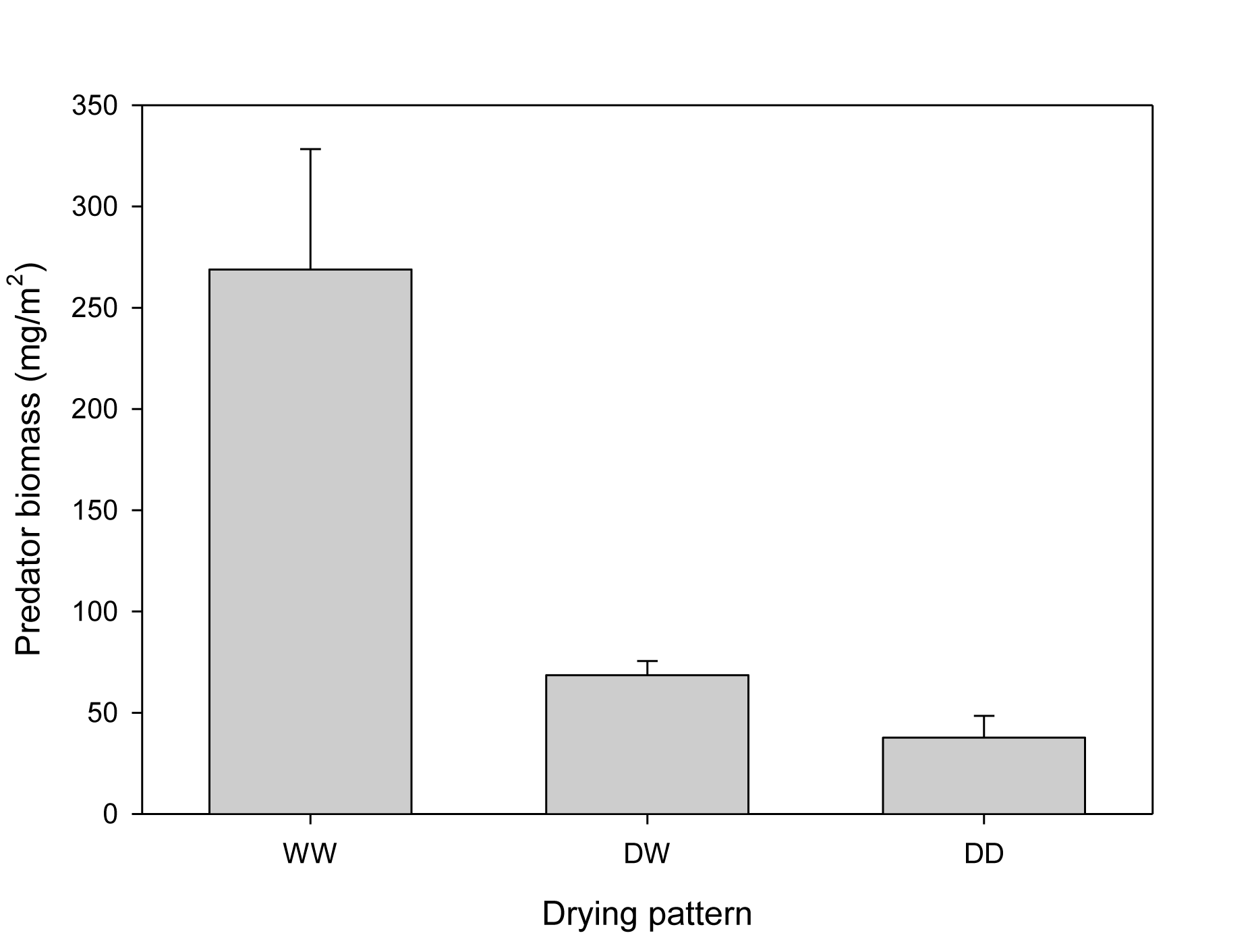

Supplement: Figure S4 — Dry weight biomass (± se) of predators in ponds inhabited by Pseudacris triseriata . Data presented as a function of pond drying pattern where WW = wet both the previous fall and the following spring; DW = dry in the fall and wet the following spring; DD = dry in fall and dry in the following spring before filling. (TIF) [file pone.0097387.s007.tif]

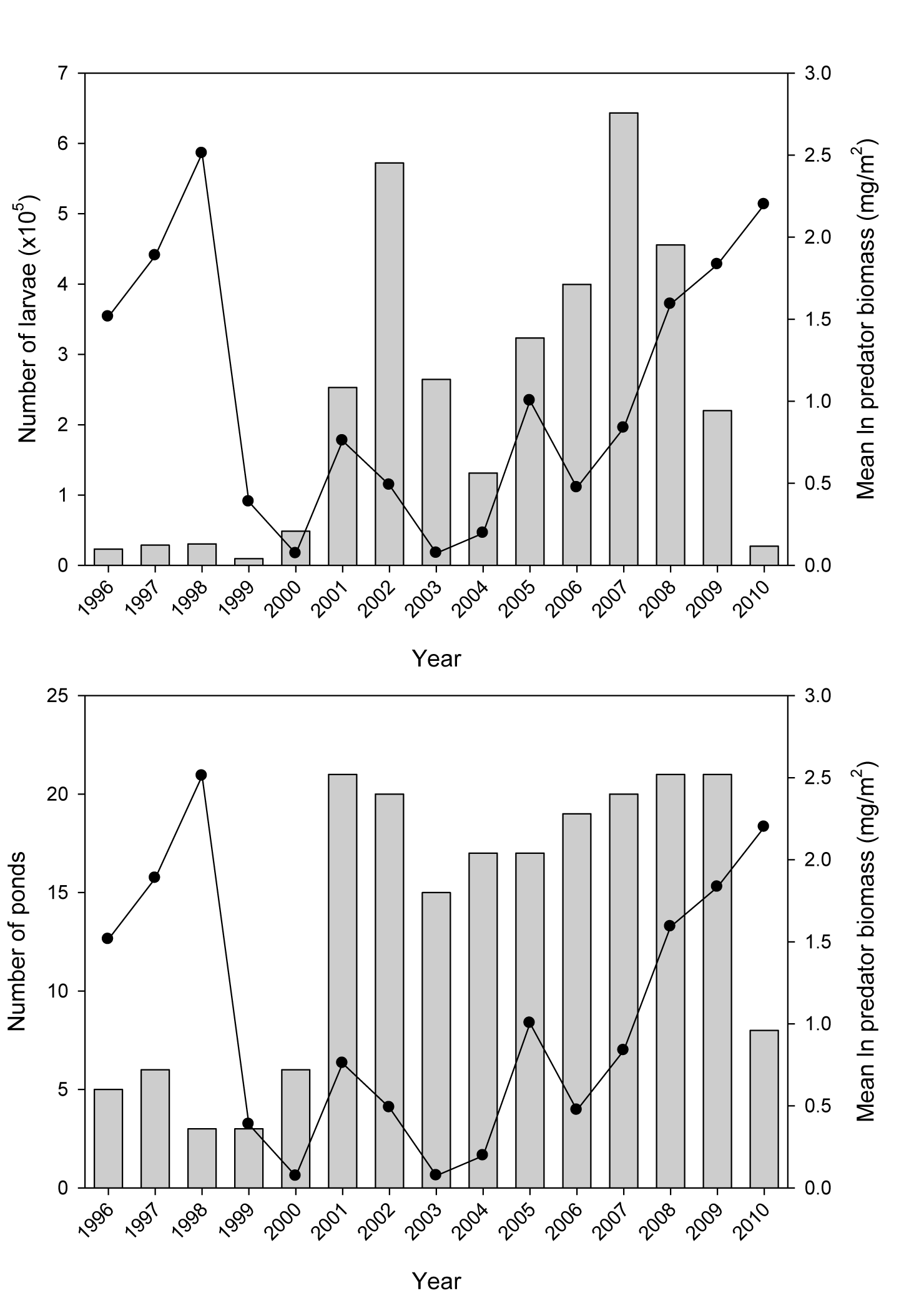

Supplement: Figure S5 — Regional population size and ponds occupied for Pseudacris triseriata across years versus average dry weight biomass of predators. Top panel is regional larval population size (histograms) and mean predator biomass in ponds (line). Bottom panel is number of ponds occupied (histograms) and mean predator biomass (line). (TIF) [file pone.0097387.s008.tif]
